# Strengthening Care for Children Using a Virtual Integrated General Practitioner–Pediatrician Model of Primary Care (SUSTAIN): Protocol for a Stepped Wedge Cluster Randomized Controlled Trial

**DOI:** 10.2196/69728

**Published:** 2026-01-14

**Authors:** Tammy Meyers Morris, Harriet Hiscock, Karen Wheeler, Corin Miller, Nan Hu, Shukri Hassan Shire, Carmen Crespo-Gonzalez, Susan Bullock, Natalie Taylor, Douglas Boyle, Lena Sanci, Kenny Lawson, Louisa Adams, Ken Peacock, Michael Hodgins, Annemarie Christie, Raghu Lingam

**Affiliations:** 1 Population Child Health Research Group, Discipline of Paediatrics & Child Health, School of Clinical Medicine, University of New South Wales, Building C29 HTH Level 3, 55 Botany St Kensington, Sydney, NSW, 2033, Australia, 61 0452494461; 2 Health Services and Economics, Murdoch Children's Research Institute, Melbourne, Australia; 3 Department of Paediatrics, University of Melbourne, Melbourne, Australia; 4 Implementation to Impact, School of Population Health, University of New South Wales, Sydney, Australia; 5 Department of General Practice and Primary Care, University of Melbourne, Melbourne, Australia; 6 Translational Health Research Institute, School of Medicine, Western Sydney University, Sydney, Australia; 7 Faculty of Medicine and Health, School of Clinical Medicine, University of New South Wales, Sydney, Australia; 8 Sydney Children’s Hospitals Network, Sydney, Australia

**Keywords:** general practice, pediatrics, randomized controlled trial, virtual integrated care model, health services, primary care

## Abstract

**Background:**

Australia’s health care system is under pressure. Pediatric referrals to public hospital emergency and outpatient departments have increased recently, overburdening emergency services and resulting in extended waiting times for nonurgent pediatric care. Children living outside metropolitan areas are disproportionately affected. Integrated models of care with pediatricians collaborating with general practitioners (GPs) in their practices have been evaluated in the United Kingdom and Australia. Results are promising for quality of care improvement and reducing referrals to hospitals. GPs and pediatricians found the model feasible, knowledge- and confidence-boosting. In-person pediatric-GP support is resource-intensive, limiting scalability and sustainability.

**Objective:**

The SUSTAIN trial is designed to evaluate a digitally delivered, integrated GP-pediatrician model of care. The primary objective is to determine whether the SUSTAIN model reduces GP referrals to hospital emergency departments for children <18 years. Secondary objectives include whether the model improves the delivery of guideline-concordant pediatric care by GPs, enhances GP confidence, and strengthens family trust in primary care. The trial also examines barriers and enablers to the implementation and includes a health economic evaluation comparing intervention costs with standard GP care.

**Methods:**

SUSTAIN uses a stepped wedge cluster randomized controlled trial design to implement a GP-pediatrician integrated model of care delivered digitally. Participating GP practices across metropolitan and nonmetropolitan New South Wales are included and randomly assigned a start time. The intervention consists of 12 months’ access to the shared GP-pediatrician consulting sessions with patients younger than 18 years conducted by telehealth, virtual pediatrician–led case discussions, phone/email pediatrician support, and complimentary access to the internationally renowned Sydney Child Health Program learning platform. GP and family surveys are collected at baseline and in the final month of intervention. An implementation evaluation using focus group discussions is conducted with each practice during the intervention and optional GP and family interviews at the end of the intervention. A health economic evaluation will explore the cost-effectiveness of this model of care.

**Results:**

The trial is supported through a 2.5-year New South Wales Ministry of Health Translational Research Grants Scheme. Human Research Ethics Committee approval was obtained in November 2022, and practice recruitment began in March 2023. Data collection commenced for all participating practices from September 1, 2023, with anticipated completion on February 28, 2025. Data analysis will commence from March 2025, with results expected in the first quarter of 2026.

**Conclusions:**

Positive outcomes for the SUSTAIN trial, demonstrating that virtual pediatric support for GPs in both metropolitan and nonmetropolitan areas can strengthen pediatric primary care provision, have the potential to influence future health policy. This innovative approach to integrated care could be rolled out across Australia and other countries with primary care–led health care systems facing similar challenges.

## Introduction

### Background

Demands on children’s tertiary health services in Australia and other high-income countries are growing [1]. Across Australia, children aged <15 years represent 26% of all lower urgency emergency department (ED) presentations and the highest presentation rates at ED [2]. While low urgency conditions do not necessarily equate with low severity or complexity [2], up to 74% of such presentations could be appropriately managed in a timely and safe manner within primary care [3]. High costs are involved in the hospitalization of children, both for governments and consumers. Alternative ways to strengthen pediatric primary care could avert some of the costs implicated in low-acuity presentations to hospitals [4,5].

Australia’s health care system is centralized around primary care provided by general practitioners (GPs). Over time, GPs have seen a diminishing proportion of pediatric cases, reducing their confidence in managing common pediatric conditions [6,7]. Adherence to clinical practice guideline recommendations by GPs has also been found to be reduced for some common conditions [8,9]. As the burden and complexity of disease evolve in high-income settings, increasingly, children present to general practice with medical complexity, including chronic diseases, neurodevelopmental, behavioral, and mental health issues [10-12]. The most common health system concerns expressed by GPs in Australia include an unsustainable workload in the context of this increasing patient complexity and difficulty navigating a fragmented health system [12]. These pressures impact the delivery of high-quality care, highlighting the need to better support GPs in fulfilling their vital role [13]. Challenges faced at the primary care level contribute to health inequities, especially for children living in rural and remote Australia, who experience reduced access and longer waitlists for specialist health services, contributing to diagnostic and treatment delays, adversely impacting health outcomes, and increasing health care costs [14,15].

The need for service redesign and exploration of new approaches to health care delivery for children, young people, and families has been identified to effectively manage health service demands. The primary care workforce requires support to improve care pathways for children, especially those with medical complexity and living in nonmetropolitan areas. Telemedicine and innovative technologies to support the sharing of clinical information have been highlighted as a mechanism to address this need [16].

Our group has implemented and evaluated an integrated GP-pediatrician model of care through several trials: the Strengthening Care for Children (SC4C), based on the UK Children and Young People’s Health Partnership [17], and Connecting Care for Children studies. These studies aimed to support GPs to strengthen the delivery of pediatric care, reducing unnecessary tertiary health service use and improving quality of care at the primary care level. In the UK setting, the intervention was implicated in a reduction in new patient hospital appointments, specialty referrals, and ED attendances, which was reported as a result. Families reported an increasing preference to see their GP, whilst GPs reported improved pediatric knowledge and understanding of how to navigate hospital services [18]. In Australia, the SC4C study, using an in-person, GP-pediatrician engagement model with pediatricians supporting GPs in their practices, demonstrated a reduction in referrals to hospital EDs and was found acceptable and suitable for GPs, pediatricians, and families [19]. However, the in-person model included considerable travel distances for pediatricians, even within metropolitan areas, posing equity and scalability challenges [20].

The evaluation design of the trial was discussed and piloted with GPs [19]. Although there was equipoise, one of the key areas discussed was the wish for all GPs to have access to the intervention. A stepped wedge randomized controlled trial (RCT) design was preferred to a 2-arm cluster RCT. The stepped wedge RCT design offers the gold standard of a cluster RCT. The advantage of a stepped wedge trial design ensures that all participating GP practices receive the intervention [21].

Building on learnings from the SC4C study, we propose to evaluate a model of integrated GP-pediatric care that uses a virtual approach to integrating GP-pediatric care, with the aim of demonstrating scalability and sustainability through a more equitably available model of care. The SUSTAIN study also evaluates the renowned Sydney Child Health Program (SCHP), a modular, web-based pediatric training program that has been developed for GPs, supported through the Sydney Children’s Hospital Network (SCHN) in New South Wales (NSW), and which has not previously been robustly evaluated [22].

### Objectives

Our primary and secondary objectives are to (1) reduce GP referrals to hospital ED and outpatient (OP) clinics for children and young people younger than 18 years and (2) improve GP pediatric care aligned with best practice (BP) guidelines, GP confidence, and increase family trust in primary care, while reducing family preference for specialist pediatrician referral.

Our implementation objective is to examine factors that help or hinder the implementation of SUSTAIN and understand factors associated with acceptability and scalability, and to assess the sustainability of the model.

Our economic objective is to assess the cost-effectiveness of SUSTAIN compared to that of standard GP care by undertaking a health economic evaluation (Table 1).

**Table 1.** Trial objectives, data sources, data collection methods, and outcomes.

Primary and secondary objectives	Data sources	Methods of collection	Period of data collection	Outcomes of interest
What is the impact of SUSTAIN on GP^a^ referral to hospital OP^b^ clinics and EDs?^c^ (Primary Outcome)	Referral pop-up supported through GRHANITE technologies on GP desktop	GRHANITE data extraction of referral destination (including no referral) for each pediatric visit to participating GPs.	Collected as part of pediatric GP consults at the beginning of the control data period and throughout the intervention period.	Whether or not the GP referred the child to the hospital OP clinic or emergency department.
What is the impact of SUSTAIN on GP quality of care for common childhood conditions?	GP medical records	GRHANITE data extraction of care quality based on the measurement of the CareTrack Kids indicators.	Collected as part of pediatric GP consults at the beginning of the control data period and throughout the intervention period.	Whether or not the GP followed clinical guidelines (ie, they did not request unnecessary tests or prescriptions) for 5 common childhood conditions (ie, asthma/wheezing, bronchiolitis, constipation/abdominal pain, upper respiratory infections, and infant crying, and reflux).
What is the impact of SUSTAIN on GPs? (eg, confidence and skills in pediatric care and use of clinical guidelines)	GP web-based survey	Web-based control and intervention surveys completed by GPs via REDCap^d^.	Control surveys are collected in the month prior to the implementation commencing in each practice. Intervention surveys are collected in the last month of the implementation at each practice.	Changes in the level of confidence in pediatric care, level of knowledge and skill in navigating the health system for children, and reported use of clinical guidelines.
What is the impact of SUSTAIN on patients and family experience?	Family web-based survey	Web-based control and intervention surveys completed by families/caregivers via REDCap.	Control surveys are collected in the month prior to the implementation commencing in each practice. Intervention surveys are collected in the last month of the implementation at each practice.	Level of confidence in GP care, level of satisfaction with GP care, desire for referral to specialist care, and preference for GP or specialist review.
Health economic evaluation				
What is the cost of implementing the model of care? What is the cost- effectiveness?	Trial data and supplementary unit costings	Trial data on the model of care, health service activity, and combined with relevant unit costs.	Data will be collected throughout the model of care.	Costs of conducting the model of care compared with usual care and costs/cost offsets from changes in OP/ED referrals, MBS,^e^ and PBS^f^ (including out-of-pocket fees) compared with accessing usual care.
Implementation evaluation				
What are the aspects of the model of care that make it effective or ineffective at producing system change?	Pediatrician collected data, qualitative interviews, and web-based surveys with GPs, general practice managers and administration staff, trial pediatricians, families, and children	Consolidated framework for implementation research; qualitative interviews with families, children, and practitioners; web-based surveys. Pediatricians will collect unidentifiable data on the patient characteristics (eg, age and sex) and nature of pediatric support provided (eg, reason for consult and topic of case study discussion).	Interim qualitative data will be collected via focus groups with general practice 6 months into the model of care (iterative data collection process). Interviews with GPs, families, and pediatricians will be conducted at the end of the model of care. Web-based survey data will be collected upon completion of the model of care in each general practice; pediatrician data will be collected as part of the coconsultations, case study discussions, and phone/email support throughout the model of care.	Identify strategies for successful implementation as well as barriers and facilitators in adoption, delivery, and maintenance to inform future scaling. Describe the model of care, including number of children seen in GP-pediatrician coconsultations, reasons for coconsultations, number of and reason for phone and email support to the pediatrician, number and topic of case study discussions. Feasibility/acceptability and appropriateness of the model; adoption and fidelity to the model.
Sustainability				
Explore the sustainability and enduring effects of SUSTAIN post implementation on proportion of GP pediatric referrals to OP clinics or EDs and GP quality of care compared with preintervention GP care.	GP medical records	GRHANITE data extraction of referral destination (including no referral made) for each pediatric visit to participating GPs and of care quality based on the measurement of care provided for the 5 common childhood conditions.	Data will be collected on pediatric GP consults following completion of the model of care in each general practice (ie, once access to pediatrician support has ceased) until the end of the trial.	Whether or not the GP refers the child to a hospital OP or ED. Whether or not the GP followed clinical guidelines for 5 common childhood conditions (as above). How GP pediatric referrals (1) and quality of care (2) in the sustainability period compared with the intervention period (when the pediatrician was in the clinic). Economic evaluation results reflecting national rollout in real-world sustainable settings along with budget impact.

a GP: general practitioner.

b OP: outpatient.

c ED: emergency department.

d REDCap: Research Electronic Data Capture.

e MBS: Medicare Benefits Schedule.

f PBS: Pharmaceutical Benefits Scheme.

## Methods

### Overview

This paper reports the research protocol for the SUSTAIN trial, including how we will partner with primary care, government, and pediatric hospitals to evaluate the effectiveness and cost-effectiveness of SUSTAIN in a cohort of general practices across NSW, using a stepped-wedge cluster RCT. The trial will be conducted according to the SPIRIT (Standard Protocol Items: Recommendations for Interventional Trials) checklist. We will apply the Medical Research Council framework used for developing and evaluating complex interventions [23].

### Trial Design

**Figure 1.**
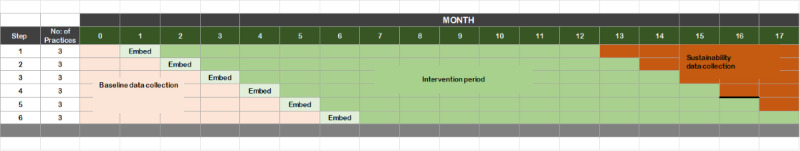
Stepped wedge randomized control trial design.

SUSTAIN is a stepped wedge cluster RCT of a virtual GP-pediatrician integrated model of care compared with standard GP care. This trial design has the rigor of a cluster RCT but allows all participating general practices to be exposed to the SUSTAIN care model, providing control, intervention, and postintervention data. This trial design was selected to ensure all practices receive the intervention [21] (Figure 1).

Each general practice provides control period data (referral and medical record data) during the control period (standard GP care, no SUSTAIN model operating). Each month thereafter, 3 practices switch concurrently from control to intervention where a pediatrician supports GPs through telehealth coconsultations, virtual case-based discussions, and being available by phone and email contact in working hours. During the first month of the intervention for each practice, a 1-month transition period is allowed to embed the SUSTAIN model of care into the practice, where data do not contribute to the analysis. This embedding period assists with implementation of the model of care into the practice and allows time to address any procedural issues. After the embedding period, each practice is exposed to an 11-month intervention period. Sustainability data are collected following cessation of the intervention until the intervention is completed for the final practice.

### Setting and Participants

SUSTAIN is a multisite trial conducted across GP practices in different local health districts within NSW. GP practices in both metropolitan and nonmetropolitan (regional, rural, and remote) areas of NSW, including areas with higher-than-average proportions of priority populations, will be eligible to participate.

Trial participants will include general practices with consenting GPs across NSW. Families of children <18 years attending the general practice may be asked to complete anonymous surveys regarding their experience of pediatric care in GP services.

Eligible general practices include those responding to an expression of interest and meeting the inclusion criteria (Textbox 1).

Textbox 1. **Trial population exclusion and inclusion criteria.**

**Inclusion criteria**

General practices:

- Be located within metropolitan and nonmetropolitan local health districts in New South Wales.
- Have best practice or medical director 3 as their electronic medical record.
- Be accredited or working toward accreditation against Royal College of General Practice standards.
- Agree to install GRHANITE software (for extracting general practitioner [GP] medical record data).

GPs:

- See patients younger than 18 years.
- Provide a minimum of 2 weeks of referral data during the control period.
- Sign participant information consent forms.

Families:

- Caregivers of children younger than 18 years who have received care at the general practice regardless of GP seen, in the prior 3 months for both the control and the intervention period.
- Caregivers with sufficient English to complete the survey.

**Exclusion criteria**

Families:

- Children or young people who present to the general practice without a parent/guardian.
- Insufficient English.

### Recruitment

#### General Practices and GPs

An expression of interest will be distributed to practice managers, GPs, and practice nurses through the primary health networks (PHNs) [24], Central and Eastern Sydney, South Western Sydney, South Eastern NSW, and directly to other general practices in rural, regional, and remote areas in NSW. Interested general practices will be required to sign a memorandum of understanding with the research team adhering to the requirements of their participation, and sign a license agreement to install the clinical data extraction software tool GRHANITE [25]. GRHANITE is discussed in the Data Management Plan section. Each GP will be required to sign a participant information consent form (Section S1 in Multimedia Appendix 1) to participate in the trial. Any GP wanting to participate during the intervention phase must provide at least 2 weeks of control referral data. Each participating practice will receive a one-off AU $1000 (approximately US $655) payment to support the implementation of the trial into their practice. An ethics-approved poster will be available for practices to promote the GP-pediatrician coconsultation service and inform patients about the study.

#### Families/Caregivers

Caregivers of patients younger than 18 years who present to a participating general practice are invited to complete an anonymous, web-based survey about their perceptions and experience of the care received during a recent GP consultation (Section S4 in Multimedia Appendix 2). These surveys will be sent by each practice via a broadcast SMS text message on behalf of the research team to families/caregivers of patients younger than 18 years who have received care at the general practice, regardless of GP seen, in the 3 months preintervention (baseline) and in the 3 months before the SUSTAIN model of care (intervention) ends. In the final survey, caregivers will be invited to voluntarily participate in a qualitative interview via telephone/web-based video led by the implementation evaluation team for the trial.

### Randomization and Blinding

Participating practices will be randomized for intervention using a predetermined web-based randomization sequence generated by an independent trial statistician. Participating general practices will be randomly ordered in terms of when they switch from standard GP care to the SUSTAIN model of care by the independent statistician. To avoid recruitment bias in clusters, randomization will occur once all general practices have been recruited and enrolled; that is, after all inclusion and exclusion criteria are addressed, and all general practices sign the relevant trial agreements. Following randomization, practices will be unblinded to their allocation status/model of care start date. It is necessary for each general practice to know their randomization position to prepare for intervention, so allocation is not concealed. It is not anticipated that GPs knowing their randomization status will impact/change their standard practice due to their demanding workload. The research team is also unblinded to each practice’s allocation status to allow them to effectively engage with each practice to prepare their implementation schedule.

### Intervention

**Figure 2.**
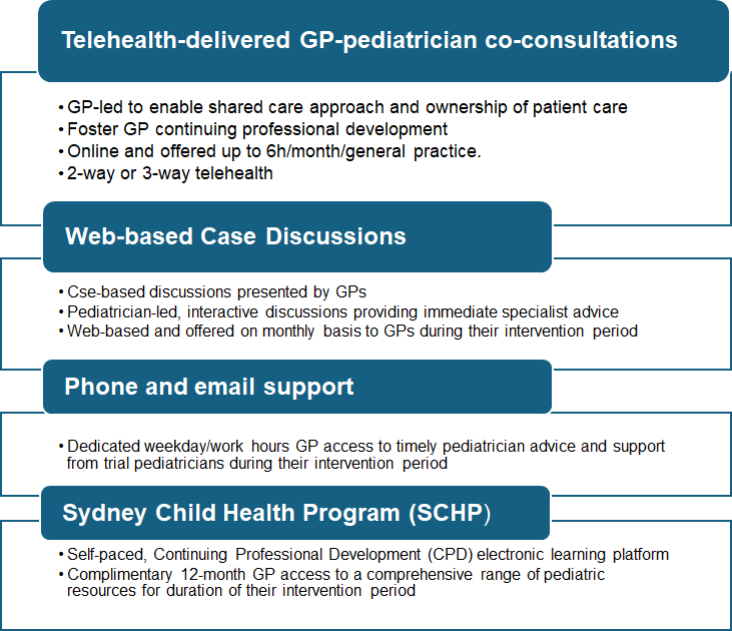
SUSTAIN intervention (model of care components). GP: general practitioner.

SUSTAIN is a 12-month-long virtual model of care offered to all consenting GPs in participating general practices. It consists of four components: (1) virtual GP-led, shared GP-pediatrician consulting sessions with patients younger than 18 years, (2) virtual pediatrician-led case discussion sessions, (3) phone/email pediatrician support, and (4) complimentary access to the internationally renowned SCHP learning platform (Figure 2).

During their 12-month intervention period, GPs may engage with any component of the model of care based on their needs, capacity, and learning style. The SUSTAN model of care will be delivered by a 1.0 FTE pediatrician (can be a shared position). The pediatricians will be employed through the SCHN.

### Data Management Plan

All participants (GPs and caregivers) will be assigned a unique numerical identifier (an ID code) generated in REDCap (Research Electronic Data Capture; Vanderbilt University) for use throughout the trial. A single electronic, password-protected database in REDCap will record all general practices, GP details, and survey data. Any data recorded will be stored on the secure University of New South Wales (UNSW) network drive on a password-protected computer and will be made available for importing into the project secure research environment. The project database will only be accessible by the designated research team members (investigators, statistician, and project managers) and hosted on the UNSW secure server, which meets security and ethical confidentiality requirements.

GRHANITE, developed and managed by the Health and Biomedical Informatics Center at the University of Melbourne (UoM), specifically designed for research purposes [25,26], extracts and curates the delivery of deidentified and encrypted primary care data to secure research data storage facilities. Person identifiers are removed from the data, in keeping with data custodian permission, and subject to ethics committee approval and legal agreement. Data extracted by GRHANITE will be stored in a Research Databank on the UoM Research Cloud, physically located within the UoM Secure Data Center. GRHANITE data will be made available to the research team from this databank via a UoM secure virtual research environment that gives researchers access to the data in a controlled manner. Upon completion of GRHANITE data extraction, only deidentified data will be transferred to the project analysis team, with a project ID unique to each patient. GRHANITE is only compatible with BP and medical director (MD) electronic medical records (EMRs), used by ≈90% of Australian general practices [27].

Patient confidentiality will be strictly held in trust by the participating investigators, research staff, and the sponsoring institution and their agents. No information concerning the trial or the data will be released to any unauthorized third party without the participant’s consent and written approval of the sponsoring institution.

All interview data will be transcribed and deidentified for analysis and will be stored in a restricted-access folder on UNSW network drives on a password-protected computer. Any personal information or quotes attributed to individual participants in published form will be anonymized.

### Data Collection

#### GP EMR Data Collection

To measure the primary outcome (ie, referrals to hospitals), pediatric referral data will be routinely collected from the GP EMRs via GRHANITE. This software will be remotely embedded into all participating GP medical software computers (compatible with BP or MD EMR software). For research purposes, GP data will be collected on all patients younger than 18 years. These data will be deidentified at the patient level, although the identity of the GP will be supplied to the research team. Collection of GP EMR referral data will commence for all practices following randomization and will continue for the duration of the trial.

The ability to record referral information, manage deidentification and consent processes, is a principal rationale for the use of the GRHANITE technologies and was demonstrated to be acceptable to GPs in the SC4C study [19].

#### GP Referrals

Data on GP referrals is not recorded in a standardized fashion in the GP EMR. Therefore, in order to measure the primary outcome, that is, referrals to hospitals, the GRHANITE team developed a tailored referral pop-up window (Section S5 in Multimedia Appendix 3) of common referral options to be completed by GPs following each pediatric consultation. This specific software extracts information regarding whether the GP referred a child and where (eg, no referral, hospital OP, or emergency department, private pediatrician, or allied health). The research team will continuously monitor GP data to promptly identify and address any technical issues posing a risk to data acquisition.

#### GP Quality of Care

GRHANITE will be used to extract deidentified patient-level data routinely collected on all patients younger than 18 years seen by consented GPs throughout the trial. Deidentified pediatric patient data will be limited to patient demographics, reason for visit, diagnoses, referrals, prescriptions, ordered imaging and pathology testing, and Medicare item billing. The patient-level data will be used to characterize GP pediatric visits (eg, number of children seen, gender of the children, and common diagnoses) and compare quality of care for common conditions before and after the model of care is implemented, based on the CareTrack Kids indicators [28].

A natural language processing algorithm, developed by the Computing and Information Systems at the UoM for the SC4C trial, will be used to automatically transform GP EMR clinical free text of “reason for visit” or diagnosis into structured clinical data, based on the Systematized Nomenclature of Medicine Clinical Terminology [29].

#### GP Model of Care Engagements

As per our ethics approval, GP engagement with the components of pediatric support, such as coconsults, case discussion, phone/email, and SCHP access, will be recorded by the following processes and stored on the secure UNSW network drive. For coconsults, GPs will record deidentified patient information (age, gender, presenting problem, and reason for coconsult) via the SCHN Microsoft Bookings. For email, phone, and case discussions, pediatricians will record via REDCap each engagement, including GP name and deidentified patient information on the topic or concern discussed. For SCHP usage, the SCHP team will record the GP name and track GP access with the SCHP learning platform.

#### GP and Family Data Collection

GP and family survey data collection will occur preintervention (baseline) and in the last month of the SUSTAIN model of care (intervention) for each practice.

A web-based GP Survey (Section S3 in Multimedia Appendix 4) will be used to measure GP experience and confidence in pediatric care, completed by all participating GPs via REDCap. Based on the SC4C trial, the survey collects information including GP demographics, factors that impact their decision to refer a pediatric patient, and knowledge and confidence in pediatric care and services. GP name and general practice will be recorded for survey completion tracking, but will not be attached to the survey responses.

A web-based family survey (Section S4 in Multimedia Appendix 2) will be used to measure caregivers’ perceptions and experience of pediatric care provided at their general practice. To provide data on a “whole of practice” level, caregivers of a patient <18 years, regardless of whether they attended a consultation with a participating GP, will be eligible to complete the Family Survey via REDCap. The survey collects information on the child/family regarding a GP visit up to 3 months prior. These surveys are voluntary, and responses are anonymous.

No power calculation has been performed, as we will recruit as many families as possible during this timeframe and use all available data.

SUSTAIN GP and family surveys have been adapted from the SC4C study and comprise items generated by the SC4C investigators and drawn from previously published literature [19,30,31]. Data collection timepoints for participant enrollment and interventions can be found in Table 2.

**Table 2.** Outline of participant enrollment, intervention, and data collection timepoints.

	Enrollment (–t_1_)	Allocation (t_0_)	Study period			Close out (t_x_)
			Control period (t_1_)	1-month Embedding period (t_2_)	11-month Intervention period (t_3_)	
Enrollment						
General practice eligibility screen	✓					
Informed GP^a^ consent	✓					
General practice intervention allocation		✓				
Intervention						
Virtual GP-pediatrician coconsultation				✓^b^	✓	
Virtual case discussions				✓^b^	✓	
Pediatrician phone/email support				✓^b^	✓	
GP access to SCHP^c^				✓^b^	✓	
Primary outcome						
GP referral outcomes						
GRHANITE extracted from routinely collected GP EMR^d^ data			✓	✓^b^	✓	
Secondary outcomes						
GPs						
Quality of care						
GRHANITE EMR data extraction on care quality based on measurement of the CareTrack Kids indicators: asthma, bronchiolitis, constipation, upper respiratory tract infections, and infant crying and gastroesophageal reflux			✓	✓^b^	✓	
Baseline and intervention /follow-up surveys						
GP demographics			✓		✓	
Confidence in pediatric care and access to pediatric services			✓		✓	
Skills to manage child health			✓		✓	
Awareness and use of HealthPathways			✓		✓	
Model feasibility, acceptability, feedback, and patient benefit					✓	
Families/caregivers						
Baseline and intervention /follow-up surveys						
Family demographics			✓		✓	
Confidence in GP care			✓		✓	
Quality of care and interactions with the GP			✓		✓	
Preference for pediatrician referral and GP review			✓		✓	
General practice implementation focus groups				✓		
Implementation evaluation						✓
Economic evaluation						✓
Reporting and manuscript reporting						✓

a GP: general practitioner.

b Data collected during the embedding period will not be analyzed.

c SCHP: Sydney Child Health Program.

d EMR: electronic medical record.

### Sample Size Calculation

In the SC4C pilot study, there was a 7% reduction in GP referrals to hospital EDs and OPs, but we have powered our trial using a conservative 4% reduction [19,32]. The sample size of 18 practices with at least 60 pediatric consultations per practice per month will have 90% power to detect a 3.7% reduction (from the baseline rate of 10%) in the percentage of children who are referred to OP clinics or EDs following their GP appointment, assuming an intracluster correlation of 0.06 (derived from the pilot data [19]) and 2-sided type I error of 0.05. With the same sample size, we will have 80% power to detect a 5.5%‐6.55% reduction in the proportion of referrals to OP or ED for our planned stratification analysis by practice rurality, GP baseline referring level, years of practice, and gender.

### Engagement/Retention Strategies

Several methods will be used to maintain general practice and GP engagement in the trial and minimize “withdrawals” or “lost to follow-up.” These will include email reminders on accessing the SUSTAIN model of care components, “touch point” meetings by the researchers with practices, and quarterly newsletters providing updates and relevant trial information.

### Statistical Analysis

We will undertake descriptive analysis to describe the GP referral rate alongside the numbers of all children seen and the numbers of children referred to OP or ED by month during the study period for each practice.

All available data from each recruited GP and family will be analyzed according to an intention-to-treat principle. For the outcomes of GP referrals identified using the GP EMR data (eg, GP referral to OP clinic or ED), we will conduct mixed effects logistic regression, fitted to data collected during the control and intervention periods. The model will include a fixed effect of group (intervention vs control) and adjust for calendar time (as a continuous variable), and random effects to allow for variation in the outcomes for 3 hierarchical levels, including GP practice, GP within each practice, and patients seen by each GP.

Secondary outcomes collected at the child consultation level will be analyzed similarly, with separate models for the 5 common childhood conditions to measure quality of care. GP survey outcomes will be analyzed using mixed effects logistic regression, again including a fixed effect for group by intervention status and calendar time, and a random effect for GP practice, GPs, and patients.

We will control for baseline levels of GP referral for each practice in the analyses, GP-level factors (eg, rurality of the practice’s location, billing type, GP gender, years of practice, and number of children patients seen per week), and patient-level sociodemographic factors (eg, age at the encounter and socioeconomic status of residence). We will also consider whether the intervention effect varies by rurality of practice and GP-level factors (eg, referring level during baseline, years of practice, and GP gender) by the inclusion of interaction terms between group (intervention vs control) and these variables in the regression models.

We will assess whether the correlation structure has been mis-specified. The primary analysis will assume an exchangeable within-cluster correlation structure, meaning that the correlation between any 2 individuals within the same cluster is considered constant. To evaluate the impact of potential misspecification, we will conduct a sensitivity analysis using a block-exchangeable correlation structure within periods. This structure assumes stronger correlations among individuals within the same period, with weaker correlations across different periods. To implement this, we will extend the random-effects model to include an interaction between time and the GP practice random effect.

Prior to analysis, we will understand the mechanisms of the occurrence of missing data in the primary and secondary outcomes. If there is a small number of missing data (<5%), complete case analysis will be presented as the primary analysis. Missing data can occur due to 2 main reasons, considering the current study design: the pop-up failure to launch due to technical issues (nonmonotone missingness), and GP or GP practices withdrawal from the trial (generally monotone missingness, though could also be instances of nonmonotone, for example, personal leave such as maternity leave and retirement). It is reasonable to assume that data missing due to technical failure or personal leave occur missing completely at random. Based on our knowledge, the main reason for GP and GP practice withdrawal is the GP leaving the participating practice. Therefore, it is reasonable to assume these missing data occur as missing at random, and we will undertake multiple imputation to account for the impact of the missing data on the outcomes. It is unlikely that data is “missing not at random (MNAR)” in the nature of this current study, that is, GPs withdraw from the study because of their referral patterns, and this cannot be accounted for by all observed data. If we identify any MNAR, we will not impute the data and will report and discuss the results with limitations and the reasons potentially responsible for the missingness.

We will conduct an intention-to-treat analysis as the primary approach to understand the effect of offering the intervention on referral patterns, under real-world conditions. This means the data will be analyzed based on the information generated from a consenting GP in a randomized GP practice, according to the intervention that is supposed to be delivered in a particular period, irrespective of whether the intervention has been implemented as planned (practices analyzed by randomized step, irrespective of actual start date). Prespecified sensitivity analyses included as-treated and per-protocol analyses, modeling approaches that incorporate the exact calendar date of intervention initiation (to address early or delayed starts), and methods to explore the influence of contamination or noncompliance (for example, complier average causal effect estimates or instrumental variable techniques where appropriate).

### Economic Evaluation

From a health sector perspective, a within-trial economic evaluation will be conducted following methodological and reporting guidelines [33-35]. The investment costs of conducting SUSTAIN (including GP training, practice administrative support, pediatrician time, consults, and case-study discussions) will be assessed relative to GP usual care using standard price lists and any patient out-of-pocket payments (including Medicare Benefits Schedule, Pharmaceutical Benefits Schedule, and Independent Hospital Pricing Authority). The costs (and cost offsets) associated with changes in study outcomes (including ED presentations and OP attendances) will then be estimated using similar methods and aligned with the statistical analysis (described above). Three forms of complementary economic analysis and metrics will then be conducted using the information generated. First, the incremental net cost per referral avoided (primary study outcome) will be generated relative to usual care to estimate cost-effectiveness. Second, a return on investment will assess (expected) net cost offsets from avoiding ED/OP relative to the investment in SUSTAIN, and changes in Medicare Benefits Schedule/Pharmaceutical Benefits Scheme use. Probabilistic sensitivity analysis will assess statistical uncertainty in both analyses and the cost-effectiveness acceptability curves generated. Third, a budget impact analysis [36] will estimate the costs of implementing the model at the national level and disaggregated by multiple potential payers (State and Commonwealth). This will inform affordability considerations and combine with wider trial findings to guide translation and sustainable implementation.

### Implementation Evaluation

#### Overview

The mixed methods implementation evaluation for SUSTAIN will be based on the methodology developed for the SC4C trial [37]. We will use the Consolidated Framework for Implementation Research (CFIR) [38] to understand the effectiveness of the SUSTAIN model in driving systems change, while also identifying contextually relevant strategies for successful implementation. Factors (ie, barriers and facilitators) identified as moderating the adoption, delivery, and maintenance will also be used to inform wider state and potentially national rollout. The mixed methods evaluation will allow us to assess implementation metrics as defined by Proctor et al [39] (Table 3).

**Table 3.** Trial implementation metrics.

	Questions addressed by each implementation factor
Acceptability	Do practitioners, parents, and children view the SUSTAIN model as agreeable?
Adoption	To what extent do practitioners and parents use the SUSTAIN model?
Appropriateness	Do stakeholders perceive SUSTAIN as relevant and useful?
Fidelity	Is SUSTAIN applied as intended? Are all component parts of the intervention delivered as planned?
Coverage	How many service users of those eligible are reached?
Cost	How much does it cost to successfully implement SUSTAIN?
Sustainability	What are the factors that will allow SUSTAIN to be scaled up further?

**Figure 3.**
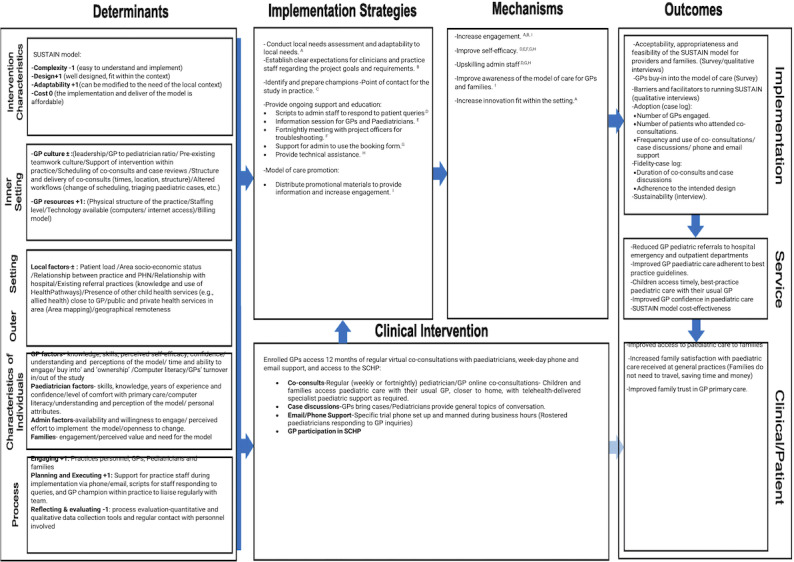
Trial logic model. GP: general practitioner; PHN: primary health network; SCHP: Sydney Child Health Program.

A logic model (Figure 3) has been developed to inform the implementation evaluation guided by the implementation research logic model [40]. The logic model encompasses the specific contextual determinants that the implementation evaluation would need to consider, both within (eg, inner context representing individual factors and organizational settings) and external to the sites (eg, area demographics and socioeconomic status). In addition to the contextual factors, the logic represents the measurable intervention characteristics, implementation strategies, mechanisms of action, and outcomes.

The implementation evaluation will use a mixed methods approach; findings will be triangulated as in our previous studies [41,42].

Surveys with GPs will be conducted at the start of and end of the intervention. GPs survey will include 2 validated instruments: the short intervention acceptability, appropriateness, and feasibility measure, and the NoMAD tool, based on the Normalization Process Theory, which assesses to what extent practitioners “buy into” SUSTAIN [43-45]. The survey will assess their experience of the current model of care and confidence in pediatric care. All GP instruments have been used in our previous SC4C trial and shown to be acceptable and feasible to use.

To determine individuals' knowledge and beliefs about the model of care, relative advantages of the model of care, GP and pediatrician self-efficacy, barriers and facilitators affecting the delivery of the intervention both from an individual and organizational perspective, the appropriateness and acceptability of the intervention; and recommendations for future implementation, we will conduct focus groups or interviews with GPs, practice managers, administrative staff, pediatricians, and families who participated in coconsultations. Interview guides have been derived from the CFIR. Practice managers and administrative staff will specifically be asked about how the model of care affected the normal operation of general practices. GPs and pediatricians will specifically be asked about features of the working relationship, for example, the collaborative nature of the relationship. Interviews with family members will determine their perceptions of the acceptability of the SC4C model and any potential adaptations to the model to make it more acceptable for families of children presenting to GP practices. Additionally, feedback from general practice staff and pediatricians will be routinely collected (eg, through meetings and email) to provide ongoing support during the implementation.

As described above, GPs will be asked to complete a short web-based survey before and after the intervention rollout via REDCap. As part of the implementation evaluation, GPs, practice managers, and administrative staff in participating practices will be contacted to participate in a focus group within each practice, with the option for an individual interview ≈6 months into the intervention and individual interviews with GPs at the end of the intervention at 12 months. Feedback will be used to determine appropriateness and acceptability of the intervention, identify barriers and enablers affecting delivery of the intervention, and sustainability of the model. The focus groups and interviews will be facilitated by investigators responsible for the implementation evaluation, and prior to the focus group or interview, the researcher will describe to participants the reasons for conducting the interview or focus group and provide a participant information sheet and a web-based consent form. The researcher will respond to any participant questions or concerns and inform participants that they can stop the interview at any time and revoke their consent to participate during or after the interview. In this event, interview recordings and transcripts will be removed from the trial and destroyed.

Family surveys conducted during the intervention period will include an item seeking permission to contact them about the opportunity to participate in a voluntary qualitative interview via telephone or web. This opportunity will only be open to families who have participated in a GP-pediatrician coconsultation, which will be determined earlier in the survey. When contacting parents or caregivers, the researcher will describe the reasons for conducting a follow-up interview, will not ask for the identity of their GP, and reassure parents that any information obtained will be confidential and will have no impact on the care their child receives from their GP. They will be asked to sign a web-based consent form.

The researcher will respond to any parent’s or caregiver’s questions or concerns and inform participants that they are able to stop the interview at any time and revoke their consent to participate during or after the interview. In this event, interview recordings and transcripts will be removed from the trial and destroyed.

Implementation evaluation data collection and analysis will assess the following.

#### Acceptability, Appropriateness, and Feasibility of the SUSTAIN Model and GPs' Buy-In Into the Model

Questionnaire data and open-ended questions from surveys will be exported into SPSS for analysis. Descriptive statistics will be calculated for all the practices recruited in the trial, including information about the inner and outer context and the intervention use and its acceptability.

#### Adoption and Fidelity

GRHANITE will allow us to access routinely collected process data from GP systems, allowing us to assess the coverage of the intervention both in terms of the number and characteristics of enrolled GPs, and the number and characteristics of children seen. This is especially important if, as we hypothesize, SUSTAIN will increase access to care for children with increased socioeconomic need. In addition to best describing the model of care, unidentifiable data will be collected on patient characteristics (eg, age and sex), reason for coconsult, topic or concern discussed at case-based discussions, and phone/email support during the 12-month intervention period at each site. This information will be recorded in REDCap and a secure UNSW network drive on a password-protected computer. No identifiable information will be collected, and data will only be analyzed to provide information on patterns of support provided. No data on individual children will be reported. This data will also provide data to understand if the intervention was implemented with fidelity in terms of the number of coconsults offered and the number of case discussions completed.

#### Barriers, Facilitators, and Strategies for Scaling Up

The CFIR domains allow us to assess not only intervention components but also the context in which primary care is currently situated. The CIFR will inform the development of the interview guides. Purposive sampling will be used to recruit a diverse sample of families, caregivers, and frontline practitioners, including GPs, pediatricians, and practice managers. Data collection for the qualitative interviews will continue until data saturation (ie, when no new themes pertaining to the research objectives are identified with subsequent interviews). All interviews and focus groups will be audio recorded and transcribed verbatim using Otter (Otter.ai, Inc), a secure transcription software. The data collected will be deidentified in preparation for data analysis. Transcripts will be analyzed thematically using an iterative thematic analysis following Braun and Clarke’s [46] approach. All participant interview audio recordings will be destroyed upon completion of the trial. Qualitative software (Nvivo 12; Lumivero) will be used to organize and classify data into emerging themes.

#### Sustainability

To assess the longer-term impact of the SUSTAIN model, sustainability data, referral data, and quality of care indicators will be collected from the time the initial practices complete the intervention. This data will be analyzed in conjunction with qualitative data collected from GPs and practice staff to assess whether changes in referral practices, adherence to standard practice guidelines, and GP confidence are sustained after the intervention has ceased.

### Ethical Considerations

This trial is approved by the Human Research Ethics Committees of SCHN (2022/ETH02068), NSW, Australia. The trial has been registered on the Australian New Zealand Clinical Trials Registry (ANZCTR number ACTRN12623000543684).

Written informed consent is obtained from each GP prior to participating in the SUSTAIN trial (Section S1 in Multimedia Appendix 1). All participants in focus groups or interviews (GPs, practice managers, administration staff, and pediatricians) are asked to complete a web-based consent form via REDCap (Section S6 in Multimedia Appendix 5).

A waiver of consent to collect deidentified data on GP care provided during a GP-pediatrician coconsultation was approved (2022/ETH02068).

Informed consent is obtained from families participating in the family experience surveys by agreeing to this through the web-based survey via REDCap. All families are provided with a plain-language statement about the study, explaining the purpose of the study and offering an opportunity to voluntarily participate in the anonymous survey (Section S2 in Multimedia Appendix 6). If the caregiver agrees to take part in the survey, the caregiver’s consent will be indicated via a checkbox at the start of the web-based survey.

Follow-up family surveys will include an item seeking their permission to be contacted about an opportunity to participate in a telephonic or web-based (eg, via Zoom and Teams) interview. Prior to conducting interviews with families, the researcher provides a participant information sheet and consent form for families to consent via web if they agree to participate (Section S7 in Multimedia Appendix 7). The REDCap e-Consent Framework provides a standardized tool to obtain consent and store consent, which automatically generates a “hard-copy” PDF of the signed forms.

Participant confidentiality will strictly be held in trust by the investigators, research staff, and the sponsoring institutions and their agents, and will be extended to cover clinical information relating to participants. The trial protocol, documentation, data, and all other information generated will be held in strict confidence and in password-protected electronic files. No information concerning the trial or the data will be released to any unauthorized third party without prior written approval of the sponsoring institutions. Investigators will have access to the final dataset via permissions maintained by the data managers.

GP practices will receive a one-off AU $1000 (US $655) payment to support implementation of the trial into their practice. GPs and families are not otherwise compensated.

The chief investigators (CIs) will maintain overall accountability, and a project manager and research assistant will provide support and assistance with trial implementation and address any process issues through the duration of the trial. Project team (weekly), research team (monthly), and advisory committee (quarterly) meetings will be held to foster information sharing, problem-solving, and decision-making regarding the trial, as well as ongoing consideration of knowledge translation. The project team includes CIs, trial pediatricians, project manager, research assistant, and implementation evaluation team; research team, in addition to project team, includes economic evaluator, trial statistician, GRHANITE representative; advisory committee, in addition to project and research teams, includes GPs, consumers, and policymakers.

CIs will hold the primary responsibility for publication of the results of the trial in accordance with the trial publication and dissemination plan. The findings from this trial will be reported according to the CONSORT (Consolidated Standards of Reporting Trials) statement guidelines [47].

## Results

The trial is supported through a 2.5-year NSW Ministry of Health Translational Research Grants Scheme (TRGS Round 6). This includes direct funding for research staff, as well as in-kind contributions from the SCHN supporting 1.0 FTE pediatrician, estimated to be the appropriate allocation of pediatrician time for the number of practices from our previous experience with the SC4C study. GPs also receive complimentary enrollment in the SCHP, a pediatric web-based modular learning course for GPs. In-kind contributions from PHNs, Central Eastern Sydney Primary Health Network, South Western Sydney Primary Health Network, and South Eastern NSW Primary Health Network also contributed to support recruitment and ongoing engagement with general practices in each PHN region. From March 2023, general practice and GP recruitment commenced across GP practices in metropolitan and nonmetropolitan (regional, rural, and remote) areas in NSW. Data collection then started in all consented practices from September 1, 2023, with anticipated completion by February 28, 2025. The delivery of the virtual 12-month-long pediatric-integrated GP intervention commenced in the first practices from October 2023 and will conclude in the final practices at the end of February 2025. GP and practice staff are engaged in focus group discussions as part of the implementation evaluation midway through the intervention phase. Data analysis, report, and manuscript preparation will begin from March 2025, with results expected to be available by the first quarter of 2026.

## Discussion

### Principal Findings

One method to prevent rising pediatric attendance at emergency departments and OP services could be strengthening the quality of pediatric health care provided by GPs closer to where people live and by increasing family confidence in the care provided by GPs for their children. This is particularly important in rural areas of countries like Australia due to the vast geographical regions. The further primary care providers are located outside of metropolitan centers in Australia, the fewer specialist services are available [48].

A primary care workforce operating at the top of the scope of practice is key to maintaining appropriate levels of services within the health care system, regardless of location. The recently released “Unleashing the Power of our Health Workforce—a Scope of Practice Review” reports that almost all health professionals in the primary care sector in Australia face some restrictions or barriers to working at the full scope of practice [49]. The SUSTAIN model, using a virtual care platform to bring GPs and pediatricians together, has the potential to empower GPs, wherever they are located, to play a greater role across the spectrum of pediatric health care from prevention, early diagnosis, management of common problems, through to chronic disease management. For children, this could reduce the need for families to seek specialist referrals for routine issues, potentially saving health care costs and wait times and leading to better long-term health outcomes. GPs may also provide recommendations for interventions for children while they are awaiting pediatrician appointments, potentially averting negative consequences of prolonged nonintervention, such as in neurodevelopmental or behavioral conditions.

Using the same rigorous mixed methods approach as for SC4C, including a stepped wedge trial design, we will be able to conduct an impact, implementation, and economic evaluation of this integrated virtual GP-pediatrician model of care for children.

There are minimal anticipated risks to patient care, as opposed to the potential benefits, with this integrated GP-pediatrician model of care proposed. However, since the model relies on relationship development between the pediatricians and GPs, it is feasible that personality issues may surface with potential negative consequences to the strengthening of confidence and skills that this model aims to enhance in primary care providers. The use of telehealth and virtual modalities for the development of these relationships may also be a challenge, as opposed to in-person models. These difficulties will be explored particularly through the implementation evaluation, where GPs, practice staff, and pediatricians are involved in focus group discussions or interviews to uncover these underlying issues. Using digital tools for health and education delivery has become more technologically stable and acceptable since the COVID-19 pandemic, and in-person modalities are less affordable and scalable. Although the estimated time available for the pediatrician to provide support for the GPs is based on prior projects, it is conceivable that this is under or overestimated. If underestimated, GPs may find it difficult to obtain the support required to assist with behavior change in practice. If overestimated, this could have financial implications for underused specialist time. These issues will be evaluated carefully, both quantitatively and qualitatively, as well as through the economic evaluation, to determine the most cost-effective way of delivering the intervention. The voice of consumers (patients/families) is obtained through anonymized surveys at baseline and postintervention, with an invitation to patients/families to voluntarily participate in an interview to gather their views on pediatric care provided at GP practices that have participated in the SUSTAIN trial.

### Limitations

The stepped wedge design is susceptible to trends over calendar time, and delay in the implementation of the model could potentially decrease motivation to participate and subsequently increase withdrawal, although researchers will maintain regular contact with prospective practices through regular “touch points,” newsletters, and emails to support motivation and engagement. Results may not be generalizable due to the general practices self-selecting to participate. In a real-world setting, cooperation of GP practices in such a study is crucial to engagement, and we actively target recruitment of a diverse range of practices through PHNs and GP networks, aiming to reach GP practices that care for underrepresented or harder-to-reach population groups, for example, remote, regional, rural general practices. General practices that only use BP or MD EMR software are included for compatibility with GHRANITE, although these EMRs are used by ≈90% of general practices in Australia [27]. Extracted data that are collected from GP EMR may not represent the true scope of care provided by the GPs who also make notes in free text, which could provide a more comprehensive assessment of care quality provided. Nevertheless, the GHRANITE data extraction tool provides a robust mechanism and is the only one of which we are aware that can extract useful data from GP practices in Australia. The voice of consumers relies on voluntary agreement to respond to pre- and postintervention surveys as well as to participate in a postintervention interview. The voice of consumers is important, and we recognize the inherent risk posed by poor participation. Nevertheless, participants are aware that this is voluntary, and their data are provided anonymously. Budget constraints also have meant that family surveys are restricted to English-speaking populations, limiting the generalizability of our findings outside these groups. To highlight the consumer voice, in further implementation and scale-up of this integrated model of care, consumer engagement will need to be a focus, including prioritizing non–English-speaking and other priority population consumers. Financial constraints also limit the collection of sustainability data beyond the period post intervention, which may underestimate the ongoing need for support between GPs and pediatricians in an ongoing manner.

### Conclusions

This study provides the potential to deliver quality pediatric care for patients across diverse settings and closer to where they live, in an equitable and scalable fashion. If this innovative integrated care approach is found to be effective and cost-effective, this could be adapted in different settings across Australia and beyond.

## Supplementary material

10.2196/69728 Multimedia Appendix 1 GP participation sheet and GP consent form. GP: general practitioner.

10.2196/69728 Multimedia Appendix 2 Family survey control and intervention period.

10.2196/69728 Multimedia Appendix 3 Sustain Referral popup box.

10.2196/69728 Multimedia Appendix 4 GP survey control and intervention period. GP: general practitioner.

10.2196/69728 Multimedia Appendix 5 GP, pediatrician, practice manager, staff interview information sheet, and consent. GP: general practitioner.

10.2196/69728 Multimedia Appendix 6 Family survey plain-language information sheet.

10.2196/69728 Multimedia Appendix 7 Family interview information sheet and consent.

10.2196/69728 Checklist 1 SPIRIT checklist.

## Abbreviations

BP: best practice
CFIR: Consolidated Framework for Implementation Research
CI: chief investigator
CONSORT: Consolidated Standards of Reporting Trials
ED: emergency department
EMR: electronic medical record
GP: general practitioner
MD: medical director
MNAR: missing not at random
NSW: New South Wales
OP: outpatient
PHN: primary health network
RCT: randomized controlled trial
REDCap: Research Electronic Data Capture
SC4C: Strengthening Care for Children
SCHN: Sydney Children’s Hospital Network
SCHP: Sydney Child Health Program
SPIRIT: Standard Protocol Items: Recommendations for Interventional Trials
UNSW: University of New South Wales
UoM: University of Melbourne
