# Supplementary material for: Strengthening Care for Children Using a Virtual Integrated General Practitioner–Pediatrician Model of Primary Care (SUSTAIN): Protocol for a Stepped Wedge Cluster Randomized Controlled Trial
Source: JMIR Res Protoc. 2026 Jan 14;15:e69728. doi: 10.2196/69728 (PMC12808869; doi:10.2196/69728)
Supplement: Multimedia Appendix 1 [file resprot-v15-e69728-s001.pdf]

# SUSTAIN GP Participant Information Sheet

The Sydney Children's Hospitals Network Human Research Ethics Committee

(SCHN HREC 2022/ETHO2068 )

| | |
|---|---|
| **Study Title** | SUSTAIN Strengthening Care for Children (SUSTAIN) |
| **Principal Investigator/s** | Professor Raghu Lingam, Professor and Financial Markets Chair in Paediatric Population Health UNSW<br><br>Dr Annemarie Christie, Director Sydney Child Health Program, SCHN |
| **Main Study Contact Person** | Tammy Morris,<br>Research Associate<br>School of Clinical Medicine<br>University of New South Wales (UNSW)<br>Email: tammy_meyers.morris@unsw.edu.au<br>Phone: 0452494461 |
| **PHD candidate** | Corin Miller<br>School of Clinical Medicine, UNSW<br>**Email**: corin.miller@unsw.edu.au<br>**Phone**: 0414980432 |

## 1. Introduction

You are invited to take part in a research study titled **SUSTAIN.** This study will be conducted in selected GP practices in three areas of the Primary Health Network (PHN); Central and Eastern Sydney PHN, Southwestern Sydney PHN (SWSPHN), South Eastern NSW PHN (SENSWPHN) and other regional, rural and remote practices in NSW, in conjunction with the Population Child Health Research Group at UNSW and the Sydney Child Health Program, Sydney Children's Hospital Network.

This informed consent sheet tells you about the study. It explains the processes involved with taking part. Knowing what is involved will help you decide if you want to take part in the study. Please read this information carefully. Ask questions about anything that you don't understand or want to know more about.

Participation in this research is voluntary. If you do not wish to take part, you do not have to.

## 2. What is the purpose of this study?

This research project aims to strengthen care for paediatric patients in primary care while decreasing referral rates to outpatient clinics and emergency departments through a new model of care. The SUSTAIN model has been previously implemented in a pilot study and found to improve family trust in and preference for GP care, and increase GP confidence in paediatric care, and reduce GP referrals to specialist paediatric services. A large multicentre study using the same model is currently underway in NSW and Victoria funded through an NHMRC-partnership grant. These studies were based on an in-person model with challenges to sustainability and equity for practices that serve priority populations and for those who are located more remotely.

The SUSTAIN model consists of: virtual GP-Paediatrician co-consulting sessions, monthly online case study discussions with clinical staff at the GP practice, and email/telephone support provided by

paediatricians to GPs in between. In addition, participating GPs will enrol in the Sydney Child Health Program, a virtual modular paediatric education course designed for GPs

The project will be undertaken within practices, in Southwestern Sydney, Central and Eastern Sydney and South Eastern NSW, as well as other regional, rural and remote areas in NSW.

## 3. Why have I been invited to participate in this study?

You are invited to take part in this research project, which is called SUSTAIN. You have been invited because the SUSTAIN study is testing a new model of care aiming to strengthen GP confidence and knowledge in paediatric care and reduce paediatric referrals to hospital Emergency Departments and Outpatient clinics. The model seeks to do so through the development of an educational relationship between GPs and the Paediatricians from the Sydney Children's Hospital Network (in New South Wales). The SUSTAIN project offers an exciting opportunity to partake in an evidence-based research intervention that aims to strengthen your paediatric skillset through professional collaboration. The GP practice you work for has agreed to participate and as a GP in the practice, you have the opportunity to participate in the trial.

## 4. Do I have to take part in this study?

Participation in any research project is voluntary. If you do not wish to take part, you do not have to. If you decide to take part and later change your mind, you are free to withdraw from the project at any stage.

Your decision whether to take part or not to take part, or to take part and then withdraw, will not your impact your relationship with professional staff or your relationship with UNSW or SCHN.

## 5. What does participation in this study involve?

The SUSTAIN model will be implemented in your practice for 12 months. You will not be asked to travel outside of your practice for your participation in this project.

As a participating GP in this project you will be asked to:

1. attend education and training events as required
2. record reason for visit and referral activities for all children you see (<18 years) in your existing electronic medical record
3. seek specialist Paediatrician advice via email and phone services as needed during weekday hours
4. attend patient co-consultations with the study Paediatrician at your practice
5. Enrol in the Sydney Child Health Program (previously DCH) at no extra cost
6. attend and present paediatric cases at monthly 60-minute virtual case study discussions, and keep information shared about patients during the multi-case discussions confidential within the discussion group
7. complete and return project surveys which evaluate your confidence in paediatric practice before and at the end of the intervention. These questionnaires are expected to take 15-20 minutes to complete.
8. Agree to have non-identifiable data extracted on all children you see (<18-years), including provider number, patient demographics (e.g., sex, age, postcode), reason for visit, prescriptions, testing ordered, and referral information.

## 6. What are the possible risks and disadvantages of taking part?

There are no expected risks, side-effects and/or discomforts in participating in this project. You may experience some inconvenience in completing the components required of your participation in the project. However, the study team will ensure they offer adequate support to lessen any inconvenience in completing these components.

Although there are no expected negative outcomes for participation in this project, if any unforeseen or unknown events arise during the undertaking of this project the study team will ensure that participants are informed and sufficiently supported.

## 7. What are the possible benefits of taking part?

Participation in this project will provide you with the opportunity to strengthen your paediatric care through collaborative education with a paediatrician. Participating GPs will have opportunities to fulfil RACGP and/or ACRRM CPD requirements (hrs) by participating in SUSTAIN model of care Your participation in this project will provide useful information for assessing the efficacy of the SUSTAIN model as a tool to strengthen paediatric care at the primary health care level, across urban, regional, rural and rural settings.

## 8. What will happen to my information?

Your name and the practice in which you work will be recorded by the study team for the purpose of tracking who has completed the online surveys but will not be attached to the survey responses to ensure that your responses are non-identifiable.

The surveys you complete will be entered into REDCap. REDCap is a secure, web-based application for building and managing online surveys and databases, developed by Vanderbilt University. REDCap is hosted on UNSW infrastructure and is securely maintained. All data transmissions between users and the REDCap server are encrypted. The study team, as well as the Ethics Committee, will have access to this information for research related purposes only.

At the conclusion of the study, all identifying participant data will be destroyed and final study data will be stored in a non-identifiable format. As per the Australian Code for the Responsible Conduct of Research, study records and non-identifiable data will be kept for a minimum of 15 years following the completion of the study, after which time the records may be kept indefinitely or destroyed.

If you withdraw from the study, we will not collect any more information about you. We would like to keep the information we have already collected about you to help us ensure that the results of the research project can be measured properly. Please let us know if you do not want us to do this.

## 9. How will the results of the study be distributed?

It is anticipated that the results of this research project will be published and/or presented in a variety of forums. In any publication and/or presentation, information will be provided in such a way that you cannot be identified, except with your expressed permission.

You can indicate on the consent form if you wish to receive a summary of the study findings.

## 10. Who should I contact if I have any questions?

If you would like more information about the project, please contact:

**Name:** Prof Raghu Lingam

**Phone:** 0433 691 232

**Email:** r.lingam@unsw.edu.au

## 11. Who do I contact if I have concerns about the study?

All research in Australia involving humans is reviewed by an independent group of people called a Human Research Ethics Committee (HREC). This study has been approved by the Sydney Children's Hospitals Network (SCHN) HREC **(approval number: 2022/ETH02068)**.

If you have any concerns or complaints about any aspect of the project or the way it is being conducted, you may contact the Executive Officer of the SCHN HREC on (02) 9845 1253 or SCHN-Ethics@health.nsw.gov.au.

## Participants-Consent

| Study Title | SUSTAIN Strengthening Care for Children (SUSTAIN) |
| --- | --- |
| **Principal Investigator/s** | Professor Raghu Lingam, Professor and Financial Markets Chair in Paediatric Population Health UNSW<br><br>Dr Annemarie Christie, Director Sydney Child Health Program, SCHN |
| **Main Study Contact Person** | Tammy Morris,<br>Research Associate<br>School of Clinical Medicine<br>University of New South Wales (UNSW)<br>**Email:** tammy_meyers.morris@unsw.edu.au<br>**Phone:** 0452494461 |

*Declaration by Participant*

☐ I have read the Participant Information Sheet or someone has read it to me in a language that I understand.

☐ I understand the purposes, procedures and risks of the research project described in the Participant Information Sheet.

☐ I have had an opportunity to ask questions and I am satisfied with the answers I have received.

☐ I freely agree to participate in this research project as described and understand that I am free to withdraw at any time during the project without affecting my relationship with SCHN or UNSW.

☐ I understand that I will be given a signed copy of this document to keep.

☐ I wish to receive a lay summary of the study findings via the following email / post address:

_______________________________________________________________

Name of Participant (please print): ______________________________________________

Signature of Participant: ____________________________ Date: ______________________

*Under certain circumstances (see Note for Guidance on Good Clinical Practice CPMP/ICH/135/95 at 4.8.9) a witness* to informed consent is required.*

Name of Witness* to Participant Signature (please print): __________________________________

Signature of Witness: __________________________________ Date: _______________________

* The Witness is not to be the investigator, a member of the study team or their delegate. In the event that an interpreter is used, the interpreter may not act as a witness to the consent process. Witnesses must be over 18 years of age
