# Supplementary material for: Strengthening Care for Children Using a Virtual Integrated General Practitioner–Pediatrician Model of Primary Care (SUSTAIN): Protocol for a Stepped Wedge Cluster Randomized Controlled Trial
Source: JMIR Res Protoc. 2026 Jan 14;15:e69728. doi: 10.2196/69728 (PMC12808869; doi:10.2196/69728)
Supplement: Multimedia Appendix 2 [file resprot-v15-e69728-s002.pdf]

Family Survey – Control Period

Your Child's Appointment at the GP Practice

About This Survey

This survey is about the care your child received at a recent appointment.
Your feedback will help us learn about your experience and how we could make it better.
It is up to you whether you want to take part - you don't have to.
You do not need to tell us your name, so please be honest - nobody will know who said what.
Your answers are confidential, and will help us to improve our service.
The survey will take about 10 minutes to complete.

Who is the Survey For?

The questions are for the parents or carers who attended the appointment with the child.

How to Fill Out the Survey

For each question, please choose one response.
You are welcome to add any further comments at the end of the survey.

Section 1: About you and your child

1.1 What is your relationship to the child you brought to a recent GP appointment?

- ☐ Mother
- ☐ Father
- ☐ Other, please specify: ________________________________

1.2 What is your child's gender?

- ☐ Male
- ☐ Female
- ☐ Other, please specify: _____________________________

1.3 How old is the child you brought to a recent GP appointment?

- ☐ 0-1 year old
- ☐ 2-5 years old
- ☐ 6-12 years old
- ☐ More than 12 years old

1.4 What is the birth order of the child you brought to a recent GP appointment?

- ☐ First born
- ☐ Second born
- ☐ Third born
- ☐ Other born

1.5 In general, would you say your child's health is:

- ☐ Excellent
- ☐ Very Good
- ☐ Good
- ☐ Fair
- ☐ Poor

1.6 For what type of concern did you bring your child to a recent GP appointment? Tick all that apply

- ☐ A long-term medical concern (e.g., asthma, diabetes)
- ☐ A short-term medical concern (e.g., fever, sore throat)
- ☐ An injury (e.g., broken bone, cut, sprain)
- ☐ A behavioural concern (e.g., tantrums, toileting, aggression, anxiety)
- ☐ A developmental concern (e.g., delay in language or motor skills)
- ☐ A routine check-up (e.g., immunisation, post-natal check-up)
- ☐ To request a repeat prescription
- ☐ To request a referral
- ☐ Other, please specify: ______________________

Branching Logic – if carer chooses a medical, behavioural, or developmental concern to Question 1.6

1.6b. Is this a new or ongoing concern for your child?

- ☐ New
- ☐ Ongoing

1.7 Is your child covered by any type of private health insurance?

- ☐ Yes
- ☐ No

1.8 What is your child's home postcode?

☐☐☐☐

1.9 Which of these is the MAIN language spoken at the child's home?

- ☐ English
- ☐ Other (please specify): _____________________

1.10 Which of the following best describes the annual income (pre-tax) of the child's household?

- ☐ $40,000 or less
- ☐ $40,001 to $65,000
- ☐ $65,001 to $90,000
- ☐ $90,001 or more
- ☐ I prefer not to say

1.11 What is your country of birth?

- ☐ Australia
- ☐ India
- ☐ Vietnam
- ☐ China
- ☐ Greece
- ☐ Italy
- ☐ Other, please specify: ______________________

1.12 How many children do you care for all together? (Including the child you brought to the GP clinic)

☐☐

1.13 What is the highest level of education you have completed? (please select one)

☐ Secondary school or less
☐ Trade or other certificate level qualification
☐ Bachelor degree
☐ Postgraduate qualification

1.14 How much time have you needed to take away from your usual duties (paid or unpaid) to seek any form of healthcare for your child in the last month?

☐☐ hours

1.15 What is your usual mode of transport when taking your child to healthcare? (please select one)

☐ Own car
☐ Public transport
☐ Taxi
☐ Walk
☐ Bicycle
☐ Other, please specify: _______________-

Branching Logic – following questions (1.18 & 1.19) to only appear if child is of school age (2 – 18 years for Question 1.3)

1.16 How much total time (approximately) in the last month has your child had off school or kindergarten to attend healthcare? Please select the response that most closely reflects your experience.

☐ None
☐ 2 hours
☐ Half a day (4 hours)
☐ A whole day (8 hours)
☐ More than 1 day

Branching Logic – is carer selected 'More than 1 day' for Question 1.18

1.16b How many days in the last month has your child had off school or kindergarten to attend healthcare?

☐☐ days

Section 2: Your thoughts about the care provided by the GP for your child at a recent appointment

Please read each statement and tick the box that best describes how confident you feel.

How confident do you feel that the GP…	Not at all confident	Not very confident	Fairly confident	Completely confident	Not applicable
2.1 … can provide general care for your child?	☐	☐	☐	☐	☐
2.2 … can manage and coordinate short and long-term care for your child?	☐	☐	☐	☐	☐
2.3 … can treat your child for the health concern for which you attended the appointment?	☐	☐	☐	☐	☐
2.4 …can provide follow-up care for the health concern for which you attended the appointment?	☐	☐	☐	☐	☐
2.5 … can share responsibility with a paediatrician (specialist in children's health) for the care of the health concern For which you attended the appointment?	☐	☐	☐	☐	☐

Please read each statement carefully, and select the box that best describes how much you agree with each statement.

	Strongly Disagree	Disagree	Agree	Strongly Agree	Not applicable
2.6 I prefer my child to see a paediatrician rather than a GP for any issue	☐	☐	☐	☐	☐
2.7 When I came to the appointment, I was hoping for a referral to see a paediatrician	☐	☐	☐	☐	☐
2.8 I take my child to the GP only when I need a referral	☐	☐	☐	☐	☐
2.9 The GP will give my child a referral to see a paediatrician whenever I ask	☐	☐	☐	☐	☐

Please read each statement carefully and select the box that best describes how you feel about each statement.

	Never	Rarely	Sometimes	Always	Not applicable
2.10 The GP listens to what I have to say	☐	☐	☐	☐	☐
2.11 The GP helps me to understand any recommendations given about my child's care	☐	☐	☐	☐	☐

2.12 I receive enough information from my GP about any questions or concerns I have	☐	☐	☐	☐	☐
2.13 The GP coordinates my child's care with other doctors	☐	☐	☐	☐	☐
2.14 The GP involves me in decisions about my child's care	☐	☐	☐	☐	☐
2.15 I receive high quality care for my child from the GP	☐	☐	☐	☐	☐
2.16 I get the care I need for my child from the GP	☐	☐	☐	☐	☐
2.17 My child's health care needs are met by the GP	☐	☐	☐	☐	☐

Section 3: Questions about follow-up care for your child following a recent appointment

3.1 Did the GP request your child return to their clinic after the recent appointment?

☐ Yes
☐ No **(branching logic skips to Question 3.2)**
☐ Unsure

3.1 a My first preference for follow up care from recent appointment is:

Follow-up at my GP Practice:

☐ with a GP
☐ with a nurse at the practice
☐ with a paediatrician (child health specialist) at the practice
☐ with a GP and paediatrician (child health specialist) together at the practice

Follow-up arranged at a hospital or another setting in the community:

☐ with a nurse
☐ with a paediatrician (child health specialist)
☐ with another specialist doctor
☐ with an allied health professional (e.g., psychologist, speech pathologist, dietitian)

3.2 If the health condition your child was here for were to worsen slightly, where would you likely first seek advice or treatment?

☐ A GP
☐ The emergency
☐ department A general
☐ paediatrician
☐ A specialist doctor
☐ Nurse-on-call or other phone/online health information service
☐ Phoning emergency services (e.g. 000, ambulance)

☐ A pharmacist or local chemist

Section 4: Final thoughts

4.1 How likely is it that you would recommend this GP practice to your friends and family?

Not at all likely ... *Very likely*

☐ 0 ☐ 1 ☐ 2 ☐ 3 ☐ 4 ☐ 5 ☐ 6 ☐ 7 ☐ 8 ☐ 9 ☐ 10

4.2 Was there anything that you thought was really good about your visit with your GP?

4.3 Was there anything that you thought could have been better about your visit with your GP ?

Family Survey - Intervention period

Your Child's Appointment at the GP Practice

About This Survey

Thank you for taking the time to complete this survey. Your GP clinic is involved in a study with Sydney Children's Hospitals Network called the SUSTAIN Strengthening Care for Children Project. This project is investigating the impact of having a paediatrician support GPs in their practice to strengthen GP care for children and adolescents.

This survey is about the care your child received a recent GP appointment.
Your feedback will help us learn about your experience, and how we could make it better.
It is up to you whether you want to take part - you don't have to.
You do not need to tell us your name, so please be honest - nobody will know who said what.
Your answers are confidential, and will help us to improve our service.
The survey will take about 10 minutes to complete.

Who is the Survey For?

The questions are for the parents or carers who attended the appointment with the child.

How to Fill Out the Survey

For each question please choose one response.
You are welcome to add any further comments at the end of the survey.

Please view the Family Survey Information Statement attached and indicate your consent below:
I have understood the information provided to me in the Participant Information and agree to participate in this survey Y/N

Section 1: About you and your child

1.1 What is your relationship to the child you brought to a recent GP appointment

- ☐ Mother
- ☐ Father
- ☐ Other, please specify: ______________________________

1.2 What is your child's gender?

- ☐ Male
- ☐ Female
- ☐ Other, please specify: ___________________________

1.3. How old is the child you brought to a recent GP appointment ?

- ☐ 0-1 years old
- ☐ 2-5 years old
- ☐ 6-12 years old
- ☐ More than 12 years old

1.4 What is the birth order of the child you brought to a recent GP appointment ?

- ☐ First born
- ☐ Second born
- ☐ Third born
- ☐ Other born

1.5 In general, would you say your child's health is:

- ☐ Excellent
- ☐ Very Good
- ☐ Good
- ☐ Fair
- ☐ Poor

1.6 For what type of concern did you bring your child to a recent GP appointment? Tick all that apply.

- ☐ A long-term medical concern (e.g., asthma, diabetes)
- ☐ A short-term medical concern (e.g., fever, sore throat)
- ☐ An injury (e.g., broken bone, cut, sprain)
- ☐ A behavioural concern (e.g., tantrums, toileting, aggression, anxiety)
- ☐ A developmental concern (e.g., delay in language or motor skills)
- ☐ A routine check-up (e.g., immunisation, post-natal check-up)
- ☐ To request a repeat prescription
- ☐ To request a referral
- ☐ Other, please specify: _

Branching Logic – if carer chooses a medical, behavioural, or developmental concern to Question 1.6

1.6 b Is this a new or ongoing concern for your child?

- ☐ New
- ☐ Ongoing

1.7 Is your child covered by any type of private health insurance?

- ☐ Yes
- ☐ No

1.8 What is the child's home postcode?

☐☐☐☐

1.9 Which of these is the MAIN language spoken at the child's home?

- ☐ English
- ☐ Other (please specify): _

1.10 Which of the following best describes the annual income (pre-tax) of the child's household?

- ☐ $40,000 or less
- ☐ $40,001 to $65,000
- ☐ $65,001 to $90,000
- ☐ $90,001 or more
- ☐ I prefer not to say

1.11 What is your country of birth?

- ☐ Australia
- ☐ India
- ☐ Vietnam
- ☐ China
- ☐ Greece
- ☐ Italy
- ☐ Other, please specify: _

1.12 How many children do you care for all together? (Including the child you brought to the GP clinic)

☐☐

1.13 What is the highest level of education you have completed? (please select one)

☐ Secondary school or less
☐ Trade or other certificate level qualification
☐ Bachelor degree
☐ Postgraduate qualification

1.14 On average, how many hours of paid work do you do per week?

☐☐ hours

1.15 How much time have you needed to take away from your usual duties (paid or unpaid) to seek any form of healthcare for your child in the last month?

☐☐ hours

1.16 How many minutes do you travel from home to attend this GP practice?

☐☐ minutes

1.17 What is your usual mode of transport when taking your child to healthcare? (please select one)

☐ Own car
☐ Public transport
☐ Taxi
☐ Walk
☐ Bicycle
☐ Other, please specify: _______________-

Branching Logic – following questions (1.18 & 1.19) to only appear if child is of school age (2 – 18 years for Question 1.3)

1.18 How much total time (approximately) in the last month has your child had off school or preschool to attend healthcare?

☐ None
☐ 2 hours
☐ Half a day (4 hours)
☐ A whole day (8 hours)
☐ More than 1 day

Branching Logic – if carer selected 'More than 1 day' for Question 1.18

1.18 b How many days in the last month has your child had off school or preschool to attend healthcare?

☐☐ days

(branching if child 2-18)

1.19 Has your child had to miss school or preschool to attend a recent GP appointment? How much time have they missed?

☐ None
☐ 2 hours
☐ Half a day (4 hours)
☐ A whole day (8 hours)
☐ More than 1 day

Branching Logic – if carer selected 'More than 1 day' for Question 1.19

1.19 b How many days has your child had off school or preschool to attend a recent GP appointment?

☐☐ days

Section 2: Your thoughts about the care provided by the GP at a recent appointment.

Please read each statement and tick the box that best describes how confident you feel.

How confident do you feel that the GP…	Not at all confident	Not very confident	Fairly confident	Completely confident	Not applicable
2.1 … can provide general care for your child?	☐	☐	☐	☐	☐
2.2 … can manage and coordinate short and long term care for your child?	☐	☐	☐	☐	☐
2.3 … can treat your child for the health concern you attended for recently?	☐	☐	☐	☐	☐
2.4 … the GP can provide follow-up care for the health concern you attended for recently?	☐	☐	☐	☐	☐
2.5 … can share responsibility with a paediatrician (specialist in children's health) for the care of the health concern you attended for recently	☐	☐	☐	☐	☐

Please read each statement carefully and select the box that best describes how much you agree with each statement.

	Strongly Disagree	Disagree	Agree	Strongly Agree	Not applicable
2.6 I prefer my child to see a paediatrician rather than a GP for any issue	☐	☐	☐	☐	☐
2.7 When I came for a recent appointment I was hoping for a referral to see a paediatrician	☐	☐	☐	☐	☐
2.8 I take my child to the GP only when I need a referral	☐	☐	☐	☐	☐
2.9 The GP will give my child a referral to see a paediatrician whenever I ask	☐	☐	☐	☐	☐

**Please read each statement carefully and select the box that best describes how you feel about each statement.**	Never	Rarely	Sometimes	Always	Not applicable
2.10 The GP listens to what I have to say	☐	☐	☐	☐	☐
2.11 The GP helps me to understand any recommendations given about my child's care	☐	☐	☐	☐	☐
2.12 I receive enough information from my GP about any questions or concerns I have	☐	☐	☐	☐	☐
2.13 The GP coordinates my child's care with other doctors	☐	☐	☐	☐	☐
2.14 The GP involves me in decisions about my child's care	☐	☐	☐	☐	☐
2.15 I receive high quality care for my child from the GP	☐	☐	☐	☐	☐
2.16 I get the care I need for my child from the GP	☐	☐	☐	☐	☐
2.17 My child's health care needs are met by the GP	☐	☐	☐	☐	☐

Section 3: Questions about who your child's appointment was with at the last visit

3.1 Who did your child have an appointment with at your last visit? (please select one)

- ☐ A GP alone
- ☐ A GP and the SUSTAIN Paediatrician
- ☐ Other, please specify: ________(skip to section 4)

3.2 Before your last appointment did you want a referral to a paediatrician for your child's issue that you sought help with from the GP?

- ☐ Yes
- ☐ No **(branching logic skips to Question 3.3)**

3.2 a After your last appointment, would you still like a referral to a paediatrician for your child's issue that you sought help with from the GP?

- ☐ Yes
- ☐ No

3.3 I was satisfied with the care my child received with my GP at the last visit

Not at all Satisfied	Not very satisfied	Fairly satisfied	Completely satisfied
☐	☐	☐	☐

3.4 Would you recommend the approach of having your GP and paediatrician working together to other families?

- ☐ Yes, please specify why: _

☐ No, please specify why: ____
____

3.5 Do you consent to a follow-up interview?

We would like to invite you to take part in an interview with our project team to further understand your experience of your GP working together with the paediatrician at this clinic. The interview will take approximately 30 minutes and be held either via phone or online video at a time convenient for you.

☐ No
☐ Yes

Please provide your full name, phone number, and email address to be contacted by the project team to arrange a time for the interview.

Please note: Your contact details will used solely for the purpose of scheduling the interview and will not be linked to your responses during analysis.

Section 4: Questions about follow-up care for your child following the last appointment

4.1 Did the GP request your child return to their clinic after the last appointment?

☐ Yes
☐ No **(branching logic skips to Question 4.2)**
☐ Unsure

4.1a My first preference for follow up care from the last appointment is:
Follow-up at my GP Practice:

☐ with a GP
☐ with a nurse at the practice
☐ with a paediatrician (child health specialist) at the practice
☐ with a GP and paediatrician (child health specialist) together at the practice

Follow-up arranged at a hospital or another setting in the community:

☐ with a nurse
☐ with a paediatrician (child health specialist)
☐ with another specialist doctor
☐ with an allied health professional (e.g., psychologist, speech pathologist, dietitian)

4.2 If the health condition your child was seen at the last appointment were to worsen slightly, where would you likely first seek advice or treatment?

- ☐ A GP
- ☐ The emergency department
- ☐ A general paediatrician
- ☐ A specialist doctor
- ☐ Nurse-on-call or other phone/online health information service
- ☐ Phoning emergency services (e.g. 000, ambulance)
- ☐ A pharmacist or local chemist

Section 5: Final thoughts

5.1 How likely is it that you would recommend this GP practice to your friends and family?

Not at all likely *Very likely*

☐ 0 ☐ 1 ☐ 2 ☐ 3 ☐ 4 ☐ 5 ☐ 6 ☐ 7 ☐ 8 ☐ 9 ☐ 10

5.2 Was there anything that you thought was really good about your last GP visit?

5.3 Was there anything that you thought could have been better about your last GP visit?
