# Supplementary material for: Strengthening Care for Children Using a Virtual Integrated General Practitioner–Pediatrician Model of Primary Care (SUSTAIN): Protocol for a Stepped Wedge Cluster Randomized Controlled Trial
Source: JMIR Res Protoc. 2026 Jan 14;15:e69728. doi: 10.2196/69728 (PMC12808869; doi:10.2196/69728)
Supplement: Multimedia Appendix 3 [file resprot-v15-e69728-s003.pdf]

Sustain(SC4C)

SUSTAIN

Please indicate below where you have referred

Mast. Albert Test

Tick all that apply

- [x] No referral

REFERRAL TO PUBLIC/COMMUNITY

- [ ] Public Hospital – Emergency Department/Direct Admission
- [ ] Public Hospital – Outpatients Department
- [ ] Public/Community Allied Health (Speech Pathology, Occupational Therapy, Audiology, Physiotherapy, Dietitian, Other)
- [ ] Public/Community Mental Health Services (eg Headspace/CAMHS)
- [ ] Early Childhood Early Intervention services (ECEI/NDIS)
- [ ] Child and Family Health Nursing, Sleep/feeding support (eg Tresillian/Karitane)

REFERRAL TO PRIVATE

- [ ] Private Paediatrician or other Non- GP specialist
- [ ] Private Allied Health (Speech Pathology, Occupational Therapy, Audiology, Physiotherapy, Dietitian, Other)
- [ ] Private Mental Health Services (Psychologist, Psychiatrist)

Save
