# Supplementary material for: Strengthening Care for Children Using a Virtual Integrated General Practitioner–Pediatrician Model of Primary Care (SUSTAIN): Protocol for a Stepped Wedge Cluster Randomized Controlled Trial
Source: JMIR Res Protoc. 2026 Jan 14;15:e69728. doi: 10.2196/69728 (PMC12808869; doi:10.2196/69728)
Supplement: Multimedia Appendix 4 [file resprot-v15-e69728-s004.pdf]

General Practitioner Survey – Control Period

About This Survey

This survey is about your experiences in providing care and making referrals for paediatric (<18 years) patients.
The survey will take about 10 minutes to complete.

How to Fill Out the Survey

For each question, please choose one response.
You are welcome to add any further comments at the end of the survey.

Name: _

General Practice: _

Your name and the practice in which you work will be recorded by the study team for tracking purposes but will not be used for analysis to ensure that your responses are de-identified

Section 1: Demographics

1.1 What is your gender?

- ☐ Male
- ☐ Female
- ☐ Other, please specify: ______________________

1.2 Are you a General Practitioner or General Practitioner Registrar?

- ☐ General Practitioner **(branching logic skips to Question 1.2b)**
- ☐ General Practitioner Registrar **(branching logic skips to question 1.3)**

1.2b For how long have you been a General Practitioner?

- ☐ Less than 6 years
- ☐ 6 – 15 years
- ☐ More than 15 years

1.3. How many half-day clinical sessions do you work per week?

- ☐ Less than 6 clinical sessions per week
- ☐ 6 – 10 clinical sessions per week
- ☐ More than 10 clinical sessions per week

1.4. What is the average number of paediatric (0 – 17 years) patients you see per week?

- ☐ Less than 11 paediatric patients
- ☐ 11 – 20 paediatric patients
- ☐ More than 20 paediatric patients

1.5. Have you ever had formal paediatric health care training outside of your MBBS, MD or GP Registrar training?

- ☐ Yes *(please specify below)*
- ☐ No

1.5b Yes (please specify) ____

1.6. What proportion of paediatric patients do you bulk bill? (please select one)

- ☐ Bulk bill all paediatric patients
- ☐ Bulk bill some paediatric patients
- ☐ Only bulk bill paediatric patients covered by a health care card/concession card (or equivalent)
- ☐ Do not bulk bill

Section 2: Importance of different issues in decision to refer

The following statements relate to your perceptions of the importance of different issues in the decision to refer a paediatric patient. Please read each question and statement and tick the box that best describes how important each statement is in your decision to refer.

2A. **How important** are each of the following **factors in your decision** to refer a child to a paediatrician?

	Very Unimportant	Somewhat Unimportant	Somewhat Important	Very Important
2.1 I have insufficient time				
2.2 The practice in which I work does not have the clinical staff necessary				
2.3 The practice in which I work does not have the necessary equipment				
2.4 It is not financially viable for the practice				

2B. **How important** are each of the following **personal factors** in your decision to refer a child to a paediatrician?

	Very Unimportant	Somewhat Unimportant	Somewhat Important	Very Important
2.5 I do not have enough knowledge about a specific child's condition				
2.6 I have no experience in treating or providing ongoing management of a specific child's condition				
2.7 I do not feel comfortable caring for a child with a chronic or complex condition				
2.8 I do not feel confident in reassuring parents that they do not need to seek a second opinion				

Section 3: Factors influencing decision to refer

The following statements relate to factors that influence your decision to refer a paediatric patient. Please read each question and statement and tick the box that best describes how often each statement influences your decision to refer.

3A. As a proportion of all of your paediatric referrals, **how often** did each of the following **factors influence your decision to refer** a child to a paediatrician?

	Rarely (<10%)	Sometimes (10-50%)	Frequently (51-90%)	Almost Always (>90%)
3.1 I wanted a second opinion to confirm a diagnosis				
3.2 I believed that a paediatrician would better manage the child's condition				
3.3 The child needed to undergo a procedure that is only provided by a paediatric specialist				

3B. As a proportion of all of your paediatric referrals, **how often** did the following **requests from a parent influence your decision to refer** a child to a paediatrician?

	Rarely (<10%)	Sometimes (10-50%)	Frequently (51-90%)	Almost Always (>90%)
3.4 A parent requested I refer their child to a paediatrician for an initial consultation				
3.5 A parent requested I renew a referral because they reported a paediatrician wanted them to return for long term care				
3.6 A parent requested I renew a referral because they wanted to continue care with a paediatrician				

Section 4: GP perspectives on the referral process

The following statements relate to your perspectives on, and experiences of, referring a paediatric patient. Please read each question and statement, and tick the box that best describes how often each statement applies to you.

4A. As a proportion of all of your paediatric referrals, **how often** have the following been **your goals for referral?**

	Rarely (<10%)	Sometimes (10-50%)	Frequently (51-90%)	Almost Always (>90%)
4.1 To receive specialist advice on a diagnosis				
4.2 To receive specialist advice on a treatment plan for a specific patient				
4.3 To receive specialist advice on episodic worsening or increasing complexity of a child's condition (e.g., exacerbation)?				
4.4 To arrange shared care with a specialist for a specific problem with a child				
4.5 For a paediatrician to take over management of a child's condition				

4B. As a proportion of all of your paediatric referrals, **how often** did you **experience** the following **outcomes from the referrals**?

	Rarely (<10%)	Sometimes (10-50%)	Frequently (51-90%)	Almost Always (>90%)
4.6 You received information (in a letter or phone call) from the paediatrician after the referral				
4.7 You considered the information you received from the paediatrician to be timely				
4.8 You considered the information you received from the paediatrician to be helpful in your management of the child's condition				
4.9 A child you referred to the paediatrician never returned to your care				

Section 5: Paediatric care and services

The following statements relate to your perspectives on, and experiences of, paediatric care and services. Please read each item, and tick the box that best describes how much you agree with each statement.

	Not at all confident	Not very confident	Fairly confident	Completely confident
5.1 I am confident I know how paediatric services are organised				

5.2 I am confident that I know how to access paediatric services for my patients				
5.3 I am confident that I have the knowledge to manage child health issues.				
5.4 I am confident that I have the skills to manage child health issues.				

Section 6: Paediatric Health Pathways

The following statements are about **Paediatric Health Pathways.** Please read each item, and tick the box that best describes how much you **agree** with each statement.

	Strongly disagree	Disagree	Agree	Strongly Agree
6.1 I am aware of what HealthPathways is				
6.2 I am aware that Paediatric Health Pathways is available to me for use within my practice				
6.3 I use Health Pathways regularly for paediatric care				
Branching logic – if participant selected Strongly Disagree or Disagree to 6.3				
6.3a I plan to use Health Pathways regularly for paediatric care				

Section 7: General practice culture

The following statements are about the culture of your general practice. Please read each item, and tick the box that best describes how much you **agree** with each statement. culture of your general practice

	Strongly disagree	Disagree	Agree	Strongly Agree
7.1 People at all levels openly talk about what is and isn't working				
7.2 Most people in this clinic are willing to change how they do things in response to feedback from others				
7.3 It is hard to get things to change in our clinic*				
7.4 I can rely on the other people in this clinic to do their jobs well				

7.5 Most of the people who work in our clinic seem to enjoy their work				
7.6 Difficult problems are solved through face-to-face discussions				
7.7 We regularly take time to reflect on how we do things				
7.8 After trying something new, we take time to think about how it worked				
7.9 People in this clinic operate as a real team				

Section 8: General practice learning climate

The following statements are about the learning climate of your general practice. Please read each item, and tick the box that best describes how much you **agree** with each statement.

	Strongly disagree	Disagree	Agree	Strongly Agree
8.1 We regularly take time to consider ways to improve how we do things				
8.2 People in our clinic actively seek new ways to improve how we do things				
8.3 This clinic encourages everyone to share ideas				
8.4 This clinic learns from its mistakes				
8.5 When we experience a problem in the clinic, we make a serious effort to figure out what's really going on				

Section 9: Intervention Appropriateness Measure

The following statements are about whether you think Strengthening Care for Children is an appropriate intervention for your practice. Please read each item, and tick the box that best describes how much you **agree** with each statement.

	Strongly disagree	Disagree	Agree	Strongly Agree
9.1 SUSTAIN seems fitting.				
9. SUSTAIN seems suitable.				

9.3 SUSTAIN seems applicable to my practice.				
9.4 SUSTAIN seems like a good match for my practice.				

Section 10: Further Comments

Any further comments about caring for paediatric patients:

Thank you for being part of the SUSTAIN Project !

General Practitioner Survey - Follow-up/Intervention Period

About This Survey

This survey is about your experiences in providing care and making referrals for paediatric patients.
The survey will take about 10-15 minutes to complete.

How to Fill Out the Survey

You are welcome to add any further comments at the end of the survey.

Name: _

General Practice: _

We only collect Name and General Practice so we can track who has completed the GP survey. Your name and General Practice will not be linked to your responses.

Section 1: Importance of different issues in decision to refer

The following statements relate to how important different issues are to you, in your decision to refer your paediatric patients. Please read each statement and select the box that best describes how important each statement is in your decision to refer.

1A. **How important** are each of the following factors in your decision to refer a child to a paediatrician?

	Very Unimportant	Somewhat Unimportant	Somewhat Important	Very Important
1.1 I have insufficient time				
1.2 The practice in which I work does not have the clinical staff necessary				
1.3 The practice in which I work does not have the necessary equipment				
1.4 It is not financially viable for the practice				

1B. **How important** are each of the following **personal factors** in your decision to refer a child to a paediatrician?

	Very Unimportant	Somewhat Unimportant	Somewhat Important	Very Important
1.5 I do not have enough knowledge about a specific child's condition				
1.6 I have no experience in treating or providing ongoing management of a specific child's condition				

1.7 I do not feel comfortable caring for a child with a chronic or complex condition				
1.8 I do not feel confident in reassuring parents that they do not need to seek a second opinion				

Section 2: Factors influencing decision to refer

The following statements relate to factors that influence your decision to refer a paediatric patient. Please read each statement and select the box that best describes **how often** each statement influences your decision to refer.

2A. As a proportion of all of your paediatric referrals, **how often** did each of the following **factors** influence your **decision to refer** children to a paediatrician?

	Rarely (<10%)	Sometimes (10-50%)	Frequently (51-90%)	Almost Always (>90%)
2.1 I wanted a second opinion to confirm a diagnosis				
2.2 I believed that a paediatrician specialist would better manage specific child's condition				
2.3 The child needed to undergo a procedure that is only provided by a paediatric specialist				

2B. As a proportion of all of your paediatric referrals, **how often** did the following **requests from a parent** influence your **decision to refer** a child to a paediatrician?

	Rarely (<10%)	Sometimes (10-50%)	Frequently (51-90%)	Almost Always (>90%)
2.4 A parent requested I refer their child to a paediatrician for an initial consultation				
2.5 A parent requested I renew a referral because they reported a paediatrician wanted them to return for long term care				
2.6 A parent requested I renew a referral because they wanted to continue care with a paediatrician				

Section 3: GP perspectives on the referral process

The following statements relate to your perspectives on, and experiences of, referring a paediatric patient. Please read each statement and select the box that best describes how often each statement applies to you.

3A. As a proportion of **all of your paediatric patients**, during your participation in the SUSTAIN project (past 12 month), **how often** have the following been your **goals for referral**?

	Rarely (<10%)	Sometimes (10-50%)	Frequently (51-90%)	Almost Always (>90%)
3.1 To receive specialist advice on a diagnosis				
3.2 To receive specialist advice on a treatment plan for a specific patient				
3.3 To receive specialist advice on episodic worsening or increasing complexity of a child's condition (e.g., exacerbation)				
3.4 To arrange shared care with a specialist for a specific problem with a child				
3.5 For a paediatrician to take over management of a child's condition				

3B. As a proportion of all paediatric advice and support you receive from the SUSTAIN paediatrician (co-consultations, phone/email/questions, lunch and learn sessions), **how often** did you experience the following?

All support and advice from the SUSTAIN paediatrician	Rarely (<10%)	Sometimes (10-50%)	Frequently (51-90%)	Almost Always (>90%)
3.6 You received information (by email, or phone/video call) from the SUSTAIN paediatrician after the referral				
3.7 You considered the information you received from the SUSTAIN paediatrician to be **timely**				
3.8 You considered the information you received from the SUSTAIN paediatrician to be **helpful in your management** of the child's condition				

Section 4: Paediatric care and services

The following statements relate to your perspectives on, and experiences of,paediatric care and services. Please read each item and select the box that best describes how much you agree with each statement.

	Not at all confident	Not very confident	Fairly confident	Completely confident
4.1 I am confident I know how paediatric services are **organised**				
4.2 I am confident that I know how to **access** paediatric services for my patients				
4.3 I am confident that I have the **knowledge** to manage child health issues.				
4.4 I am confident that I have the skills to **manage** child health issues.				

Section 5: Paediatric Health Pathways

The following statements are about Paediatric pathways in HealthPathways

Please read each statement and select the box that best describes how much you agree with each statement.

	Strongly disagree	Disagree	Agree	Strongly Agree
5.1 I am aware of HealthPathways				
5.2 I am aware that there are Paediatric pathways in HealthPathways available to me for use within my practice				
5.3 I use HealthPathways regularly for paediatric care				
Branching logic – if participant responded Strongly Disagree or Disagree to 5.3				
5.3a I plan to use HealthPathways regularly for paediatric care				

Section 6: Your experience of the SUSTAIN model of care

The following statements relate to your thoughts about the SUSTAIN model of care that you have been part of. Please read each statement and select the box that best describes how much you agree with each statement.

**Participating in SUSTAIN has…..**	Strongly Disagree	Disagree	Agree	Strongly Agree	
6.1 … strengthened my **links** with other child health professionals.					
6.2 … helped me gain **knowledge** about **how children's services are organised.**					

6.3 … helped me gain knowledge about **how to access services for my patients.**					
6.4 … increased my professional **knowledge** in child health issues.					
6.5 … increased my professional **skills** in child health issues.					
6.6 … increased my professional **confidence** in child health issues.					
**It has been feasible (practical) for me to participate in these aspects of SUSTAIN…**					
	Strongly Disagree	Disagree	Agree	Strongly Agree	Does not apply to me
6a.7 … telehealth co-consultations with the SUSTAIN Paediatrician					
6a.8 … Bi-weekly case discussions (Lunch n learn)					
6a.9 … personal 1:1 case discussion(s) with then Paediatrician (without patient present)					
6a.10 … telephone support from SUSTAIN Paediatrician					
6a.11 … email support from SUSTAIN Paediatrician					
6a. 12 Accessing the Sydney Child health Program (SCHP) online learning platform					

If you answered '*disagree or strongly disagree* to any of the questions in the previous table, can you tell us why?

As a **proportion of your referrals to the co-consulting sessions with the SUSTAIN Paediatrician**, how often were each of the following your main reason to refer a child to a co-consulting session?

	Rarely (< 10%)	Sometimes (10-50%)	Frequently (51-90%)	Almost Always (>90%)
6b.1 Education purposes – I would not typically refer this child/condition to a paediatrician, but was interested in furthering my knowledge about management and/or treatment				
6b.2 Parent reassurance – parent was keen to see a paediatrician for their child's condition				
6b.3 Warranted referral – I considered that the child needed to see a paediatrician for care				
6b.4 Other, please specify:				

6c. The following questions relate to your thoughts about the frequency of the SUSTAIN model components.

	Not often enough	Enough	Too often	I did not attend
6c.1 bi-weekly case discussions were held…				
6c.2 Telehealth co-consulting sessions with paediatricians were available/offered…				

	Strongly Disagree	Disagree	Agree	Strongly Agree
6d.1 This model of care has been beneficial for paediatric patients				
6d.2 This model of care has been beneficial for me as a GP				
6d.3 This model of care has been beneficial for the general practice I work in				
6d.4 I listened to the advice given by the SUSTAIN Paediatrician(s) with regards to care for my paediatric patients				
6d.5 I feel the SUSTAIN Paediatrician(s) listened to the advice I gave with regards to care for the paediatric patients				

6d. These questions are about how you feel the SUSTAIN model of care has had an impact for those involved

6e What do you think the impact of this model has been for your paediatric patients? (Open text)

6f What do you think the impact of this model has been for your paediatric knowledge and skills? (Open text)

6G. How have you changed your practice (the way you work) since participating in the SUSTAIN model of care? (Click apply that apply)

- ☐ Referred to a new practitioner or service - e.g. allied health, mental health services
- ☐ Utilised new resources (websites, factsheets, apps, podcasts)
- ☐ Utilised new questionnaires or assessments - e.g. ADHD questionnaires
- ☐ Other, please specify:

Please provide some examples: (open text)

Section 7: General practice culture

The following statements are about the culture of your general practice.

Please read each item and tick the box that best describes how much you **agree** with each statement. culture of your general practice

	Strongly disagree	Disagree	Agree	Strongly Agree
7.1 People at all levels openly talk about what is and isn't working				
7.2 Most people in this clinic are willing to change how they do things in response to feedback from others				
7.3 It is hard to get things to change in our clinic				
7.4 I can rely on the other people in this clinic to do their jobs well				
7.5 Most of the people who work in our clinic seem to enjoy their work				
7.6 Difficult problems are solved through face-to-face discussions				
7.7 We regularly take time to reflect on how we do things				

7.8 After trying something new, we take time to think about how it worked				
7.9 People in this clinic operate as a real team				

Section 8: General practice learning climate

The following statements are about the learning climate of your general practice. Please read each item and tick the box that best describes how much you **agree** with each statement.

	Strongly disagree	Disagree	Agree	Strongly Agree
8.1 We regularly take time to consider ways to improve how we do things				
8.2 People in our clinic actively seek new ways to improve how we do things				
8.3 This clinic encourages everyone to share ideas				
8.4 This clinic learns from its mistakes				
8.5 When we experience a problem in the clinic, we make a serious effort to figure out what's really going on				

Section 9: Intervention Appropriateness Measure

The following statements are about whether you think SUSTAIN is an appropriate intervention for your practice. Please read each item, and tick the box that best describes how much you **agree** with each statement.

	Strongly disagree	Disagree	Agree	Strongly Agree
9.1 SUSTAIN seems fitting.				
9.2 SUSTAIN seems suitable.				
9.3 SUSTAIN seems applicable to my practice.				
9.4 SUSTAIN seems like a good match for my practice.				

Section 10: Engagement & Experience

10a. For each statement, please select an answer that best suits your experience

	Strongly Disagree	Disagree	Neither agree nor disagree	Agree	Strongly Agree
10a.1 I can see how SUSTAIN differs from usual ways of working					
10a.2 Staff in this general practice have a shared understanding of the purpose of SUSTAIN.					
10a.3 I understand how SUSTAIN affects the nature of my own work					
10a.4 I can see the potential value of SUSTAIN for my work					

10b. For each statement, please select an answer that best suits your experience

	Strongly Disagree	Disagree	Neither agree nor disagree	Agree	Strongly Agree
10b.1 There are key people who drove SUSTAIN forward and got others involved					
10b.2 I believe that participating in SUSTAIN was a legitimate part of my role as a GP					
10b.3 I was open to support and collaborate with colleagues as part of SUSTAIN					

10c. For each statement, please select an answer that best suits your experience

	Strongly Disagree	Disagree	Neither agree nor disagree	Agree	Strongly Agree
10c.1 I could easily integrate SUSTAIN into my existing work					
10c.2 SUSTAIN disrupts the workflow of this general practice					
10c.3 I have confidence in my practice's ability to implement SUSTAIN in their ways of working					
10c.4 Sufficient support and resources were provided to enable staff to implement SUSTAIN					
10c.5 The practice management team adequately supported SUSTAIN					

10d. For each statement, please select an answer that best suits your experience

	Strongly Disagree	Disagree	Neither agree nor disagree	Agree	Strongly Agree
10d.1 The staff agree that SUSTAIN is worthwhile					
10d.2 I value the impacts that SUSTAIN has had on my work and my practice					
10d.3 Feedback about SUSTAIN can be used to improve it in the future					
10d.4 I can enhance my paediatric skills with SUSTAIN					

Section 11: Sustainability

11.1 Once the SUSTAIN paediatricians is no longer available at your practice, how might you maintain any acquired skills and knowledge in paediatric care?

11.2 How might new GPs in your practice be upskilled in paediatric care after the SUSTAIN paediatrician is no longer available at your practice/project has ended?

Section 12: Overall feedback

12.1 How likely is it that you would recommend this model of care to other General Practitioners?

☐ ☐ ☐ ☐ ☐ ☐ ☐ ☐ ☐ ☐ ☐

0 1 2 3 4 5 6 7 8 9 10

Not at all likely ... *Very likely*

12.2 Any further comments:

Thank you for taking the time to complete your GP survey for the SUSTAIN Project!

If you have any questions, please contact the research team at
SUSTAIN Project Team
