# Supplementary material for: Strengthening Care for Children Using a Virtual Integrated General Practitioner–Pediatrician Model of Primary Care (SUSTAIN): Protocol for a Stepped Wedge Cluster Randomized Controlled Trial
Source: JMIR Res Protoc. 2026 Jan 14;15:e69728. doi: 10.2196/69728 (PMC12808869; doi:10.2196/69728)
Supplement: Multimedia Appendix 5 [file resprot-v15-e69728-s005.pdf]

SUSTAIN Participant Information Sheet

The Sydney Children's Hospitals Network Human Research Ethics Committee (SCHN HREC)

Implementation evaluation

**Study Title**	SUSTAIN Strengthening Care for Children (SUSTAIN)
**Principal Investigator/s**	Professor Raghu Lingam, Professor and Financial Markets Chair in Paediatric Population Health UNSW<br><br>Dr Annemarie Christie, Director Sydney Child Health Program, SCHN
**Main Study Contact Person**	Tammy Morris,<br>Research Associate<br>School of Clinical Medicine<br>University of New South Wales (UNSW)<br>**Email:** tammy_meyers.morris@unsw.edu.au<br>**Phone:** 0452494461

1. Introduction

You are invited to take part in an interview for a research study titled **SUSTAIN.** This study will be conducted in selected GP practices in three areas of the Primary Health Network (PHN); Central and Eastern Sydney PHN, Southwestern Sydney PHN (SWSPHN), South Eastern NSW PHN (SENSWPHN) and other regional, rural and remote practices in NSW, in conjunction with the Population Child Health Research Group at UNSW and the Sydney Child Health Program, Sydney Children's Hospital Network.

This information sheet tells you about the study. It explains the processes involved with taking part. Knowing what is involved will help you decide if you want to take part in the study. Please read this information carefully. Ask questions about anything that you don't understand or want to know more about.

Participation in this research is voluntary. If you do not wish to take part, you do not have to.

2. What is the purpose of this study?

The SUSTAIN research project aims to strengthen care for paediatric patients provided by GPs and reduce inappropriate referrals to hospitals or other specialists. During the study, a paediatrician will work together with GPs in co-consulting sessions, monthly online case study discussions with clinical staff at the GP practice and be available to support the GPs by email/telephone. In addition, participating GPs will enrol in the Sydney Child Health Program, a virtual modular paediatric education course designed for GPs. This model of care has already been implemented with encouraging results in Victoria and NSW where paediatricians provide support in-person. In this study, an online paediatric support and education program for GPs is being trialled. The aim being to ensure that people who live anywhere can access high quality healthcare for their children close to home.

3. Why have I been invited to this study?

We have invited you to participate in an interview to understand how SUSTAIN is implemented and delivered in general practices, and to identify the local contextual differences and approaches to delivering SUSTAIN. We will ask you questions about the factors likely to impact the outcomes of SUSTAIN. We aim to capture important information at the practice, practitioner, and patient/caregiver level, which may impact the effectiveness of SC4C on outcomes. We want to know what will support and/or hinder how the SC4C model will work in general practices. We hope that knowledge gained from this process will help to inform strategies for delivering SUSTAIN on a wider scale.

4. Do I have to take part in this study?

Participation in any research project is voluntary. If you do not wish to take part, you do not have to. If you decide to take part and later change your mind, you are free to withdraw from the project at any stage.

Your decision whether to take part or not to take part, or to take part and then withdraw, will your relationship with professional staff or your relationship with UNSW or SCHN.

5. What does participation in this study involve?

We are inviting you to participate in an interview describing your experience with SC4C. The questions will be about how SUSTAIN compares to previous practice, the nature of the working relationship among participants of the SUSTAIN model, and how SUSTAIN can work better in future. The interview will take approximately 15 to 30 minutes and will be at a time and place that is convenient for you. To ensure interview responses are collected accurately, interviews will be audio recorded. Audio recordings will be transcribed verbatim and de-identified in preparation for data analysis and no results will contain any information that could identify you.

6. What are the possible risks and disadvantages of taking part?

Individuals participating in interviews may experience discomfort in different ways depending on their relationship to the study. Clinicians and staff at clinics may be uncomfortable about discussing their clinics, particularly when providing negative details about the intervention, the uptake of the intervention in the practice, or their relationship with other clinicians involved in the study. To manage these risks, all interview data will be de-identified for analysis, ensuring no identifying information is presented in outputs.

7. What are the possible benefits of taking part?

There may be no direct benefits for participants taking part in the interview.

Participation in this interview will provide you with the opportunity to describe your experience of the SUSTAIN model and provide input on the factors that supported or hindered the implementation of the model in primary care.

Your participation in this project will provide useful information for assessing and further improving the implementation of the SUSTAIN model.

8. What will happen to my information?

By signing the consent form you consent to the research team collecting and using personal information about you for the research project. Your privacy and confidentiality will be

protected at all times. Your information will only be used for the purpose of this research study and it will only be disclosed with your permission, except as required by law. For example, researchers are required to report if a participant is believed to be at risk of harm.

In order to protect your privacy, the study team will remove any information that may be used to identify you from any study documents, and instead of your name appearing on the documents, you will be identified by a specific study code number that applies only to you. Only this code number will be used on any research-related information collected about you for this study, so that your identity as part of the study will be kept completely private. Only the study team at the School of Clinical Medicine, UNSW will have the ability to link this code number with your personal information, and the linking information will be kept in on password-protected computers only accessible by the research team involved in this project. No information concerning the study or the data will be released to any third party, without prior approval by you and the written approval of the sponsoring institution. All participant interview audio recordings will be destroyed upon completion of the study. Your data will be stored for 15 years after the study finishes.

If you withdraw from the study, we will not collect any more information about you. We would like to keep the information we have already collected about you to help us ensure that the results of the research project can be measured properly. Please let us know if you do not want us to do this.

9. How will the results of the study be distributed?

It is anticipated that the results of this research project will be published and/or presented in a variety of forums. In any publication and/or presentation, information will be provided in such a way that you cannot be identified, except with your expressed permission.

You can indicate on the consent form if you wish to receive a lay summary of the study findings.

10. Who should I contact if I have any questions?

If you have any questions or want more information about this study before or during participation, you can contact:

Name: Prof Raghu Lingam

Phone: 0433 691 232

Email: r.lingam@unsw.edu.au

Name: Dr Carmen Crespo

Phone: 0406892209

Email: c.crespo@unsw.edu.au

11. Who do I contact if I have concerns about the study?

All research in Australia involving humans is reviewed by an independent group of people called a Human Research Ethics Committee (HREC). This study has been approved by the Sydney Children's Hospitals Network (SCHN) HREC **(approval number: 2022/ETH02068**.

If you have any concerns or complaints about any aspect of the project or the way it is being conducted, you may contact the Executive Officer of the SCHN HREC on (02 78251253or SCHN-Ethics@health.nsw.gov.au.

This Information Sheet is for you to keep. We will also give you a copy of the signed consent form.

Participants-Consent Form
Implementation evaluation

**Study Title**	SUSTAIN Strengthening Care for Children (SUSTAIN)

**Principal Investigator/s**	Professor Raghu Lingam, Professor and Financial Markets Chair in Paediatric Population Health UNSW<br><br>Dr Annemarie Christie, Director Sydney Child Health Program, SCHN
**Main Study Contact Person**	Tammy Morris,<br>Research Associate<br>School of Clinical Medicine<br>University of New South Wales (UNSW)<br>**Email:** tammy_meyers.morris@unsw.edu.au<br>Phone: 0452494461

Declaration by Participant

☐ I have read the Participant Information Sheet or someone has read it to me in a language that I understand.

☐ I understand the purposes, procedures and risks of the research project described in the Participant Information Sheet.

☐ I have had an opportunity to ask questions and I am satisfied with the answers I have received.

☐ I freely agree to participate in this research project as described and understand that I am free to withdraw at any time during the project without affecting my relationship with SCHN or UNSW.

☐ I understand that I will be given a signed copy of this document to keep.

☐ I wish to receive a lay summary of the study findings via the following email / post address:

____

Name of Participant (please print): ____

Signature of Participant: ___________________________ Date: _____________________

Under certain circumstances (see Note for Guidance on Good Clinical Practice CPMP/ICH/135/95 at 4.8.9) a witness to informed consent is required.*

Name of Witness* to Participant Signature (please print): ______________________________

Signature of Witness: ______________________________________ Date: ______________________

* The Witness is not to be the investigator, a member of the study team or their delegate. In the event that an interpreter is used, the interpreter may not act as a witness to the consent process. Witnesses must be over 18 years of age
