# Supplementary material for: Strengthening Care for Children Using a Virtual Integrated General Practitioner–Pediatrician Model of Primary Care (SUSTAIN): Protocol for a Stepped Wedge Cluster Randomized Controlled Trial
Source: JMIR Res Protoc. 2026 Jan 14;15:e69728. doi: 10.2196/69728 (PMC12808869; doi:10.2196/69728)
Supplement: Multimedia Appendix 6 [file resprot-v15-e69728-s006.pdf]

Participant Information Statement

HREC Project Number: 2022/ETHO 2068
Research Project Title: SUSTAIN
Principal Researcher: Prof Raghu Lingam

The University of New South Wales and Sydney Children's Hospitals Network (SCHN) are running a research project with GP practices within Central Eastern Sydney, Southwestern Sydney, South Eastern New South Wales Primary Health Networks, and other practices in rural, regional and remote areas in NSW to trial new models of care aiming to provide the support that your child needs within the GP practice with the support of paediatricians (specialists in children's health) to lessen the need for visits to the hospital for specialist care.

What are we asking you to do?

As part of this project, we are asking parents/guardians to fill out a one-off survey about their experience of care for their child from their GP. This survey will take about 10 minutes to complete and asks about your experience and feelings about the care your child has received at a recent appointment. Please try to answer all the questions. However, you are free to skip any questions you do not want to answer.

At the end of the survey you may choose to participate in an interview with our project team, to understand your experience of GPs and Paediatricians working together. The interview will take ~ 20 minutes and be held either via phone or online video at a time convenient for you. Interview transcripts will be de-identified and any quotes will be anonomyous**. Your decision to be contacted to participate in an interview is voluntary, and your views will be anonymous, and the team will not know which GP you have seen.**

We do not expect there to be any direct benefit to you or your child from filling out this survey. We hope the information will help improve care for children, and this may help other children in the future.

Who is funding this project?

This project is funded through a Ministry of Health Translational Research Grants Scheme NSW (TRGS)

What's next?

Participation in this study is voluntary and unpaid. If you do not take part, or choose to withdraw, it will not affect your access to the best available treatment options from your GP, or your care from any hospital. All survey responses are anonymous. This means that no one, including the research team, will know who has said what. Any information collected as part of this project will be treated as confidential and can only be accessed by the project research team and Sydney Children's Hospitals Network Human Research Ethics Committee

At the end of the project, we will send a summary of the results to your GP practice. You and your child will not be identifiable in any results.

If you have any questions, or would like further information about this project, please contact us.

Principal Investigator (NSW): Prof Raghu Lingam
(E): r.lingam@unsw.edu.au (P): 02 9382 5904
Or Executive Officer , SCHN HREC (02) 78251253
or SCHN-Ethics@health.nsw.gov.au.
