# Supplementary material for: Strengthening Care for Children Using a Virtual Integrated General Practitioner–Pediatrician Model of Primary Care (SUSTAIN): Protocol for a Stepped Wedge Cluster Randomized Controlled Trial
Source: JMIR Res Protoc. 2026 Jan 14;15:e69728. doi: 10.2196/69728 (PMC12808869; doi:10.2196/69728)
Supplement: Multimedia Appendix 7 [file resprot-v15-e69728-s007.pdf]

Families/Carers- Information Sheet - SCHN

The Sydney Children's Hospitals Network Human Research Ethics Committee (SCHN HREC)

Implementation evaluation

**Study Title**	SUSTAIN Strengthening Care for Children (SUSTAIN)
**Principal Investigator/s**	Professor Raghu Lingam, Professor and Financial Markets Chair in Paediatric Population Health UNSW<br><br>Dr Annemarie Christie, Director Sydney Child Health Program, SCHN
**Main Study Contact Person**	Tammy Morris,<br>Research Associate<br>School of Clinical Medicine<br>University of New South Wales (UNSW)<br>**Email:** tammy_meyers.morris@unsw.edu.au<br>Phone: 0452494461

1. Introduction

You are invited to take part in an interview for our research study titled SUSTAIN. We are also inviting children who have attended a co-consultation, so you are welcome to invite your child to attend the interview too, but this is not mandatory.

This study is being conducted in selected GP practices in three areas of the Primary Health Network (PHN); Central and Eastern Sydney PHN, Southwestern Sydney PHN (SWSPHN), South Eastern NSW PHN (SENSWPHN) and other regional, rural and remote practices in NSW,_in conjunction with the Population Child Health Research Group at UNSW and the Sydney Child Health Program, Sydney Children's Hospital Network.

This information sheet tells you about the study. It explains the processes involved in taking part. Knowing what is involved will help you decide if you want to take part in the study. Please read this information carefully. Ask questions about anything that you don't understand or want to know more about.

Participation in this research is voluntary. If you do not wish to take part, you do not have to.

2. What is the purpose of this study?

This research project aims to strengthen care for paediatric patients in primary care while decreasing referral rates to outpatient clinics and emergency departments through a new model of care. The SUSTAIN model has been previously implemented in a Pilot study and found to improve family trust in and preference for GP care, and increase GP confidence in paediatric care, and reduce GP referrals to specialist paediatric services. A large multicentre study using the same model was conducted in NSW and Victoria funded through an NHMRC-partnership grant. These studies were based on an in-person model with challenges to sustainability and equity for practices that serve priority populations and for those who are located more remotely.

The SUSTAIN model consists of: virtual GP-Paediatrician co-consulting sessions, monthly online case study discussions with clinical staff at the GP practice, and email/telephone

support provided by paediatricians to GPs in between. In addition, participating GPs will enrol in the Sydney Child Health Program, a virtual modular paediatric education course designed for GPs

The results of this research will be used by the researcher Dr Corin Miller to obtain a PHD degree.

3. Why have I been invited to this study?

We have invited you and your child to participate in an interview to understand how this new model of care is changing you and your child's experience of visiting a general practice. We will ask you questions about your experience of going to the GP. We want your opinions about what worked well and what didn't so we can improve this way of delivering health care.

4. Do I have to take part in this study?

Participation in any research project is voluntary. If you do not wish to take part, you do not have to. If you decide to take part and later change your mind, you are free to withdraw from the project at any stage.

Your decision whether to take part or not to take part, or to take part and then withdraw, will not affect your and your child's routine care, your relationship with professional staff or your relationship with the Sydney Children's Hospital network or UNSW

5. What does participation in this study involve?

If you decide to take part in this study, we are inviting you and your child to participate in an interview describing your experience with SUSTAIN. The questions will be about your experiences with the co-consultation. The interview will take approximately 30 to 40 minutes and will be at a time and place that is convenient for you, or on the phone or via videoconference. To ensure interview responses are collected accurately, interviews will be audio recorded. Audio recordings will be transcribed verbatim and de-identified in preparation for data analysis and no results will contain any information that could identify you.

6. What are the possible risks and disadvantages of taking part?

Individuals participating in interviews may experience discomfort in different ways depending on their relationship to the general practice and health professionals providing their care. Parents/guardians may be uncomfortable about discussing their clinics, particularly when providing negative details about the intervention, the take up of the intervention in the practice, or their relationship with other clinicians involved in the study. To manage these risks, any information from individual interviews will not be shared with the GPs or paediatricians looking after your child.

7. What are the possible benefits of taking part?

There may be no direct benefits for participants taking part in the interview.

Participation in this interview will provide you with the opportunity to describe your experience of the SUSTAIN model.

Your participation in this project will provide useful information for assessing and further improving the implementation of the SUSTAIN model

8. What will happen to my information?

By signing the consent form you consent to the research team collecting and using personal information about you for the research project. Your privacy and confidentiality will be protected at all times. Your information will only be used for the purpose of this research study and it will only be disclosed with your permission, except as required by law. For example, researchers are required to report if a participant is believed to be at risk of harm.

In order to protect your privacy, the study team will remove any information that may be used to identify you from any study documents, and instead of your name appearing on the documents, you will be identified by a specific study code number that applies only to you. Only this code number will be used on any research-related information collected about you for this study, so that your identity as part of the study will be kept completely private. Only the study team at the School of Clinical Medicine, UNSW will have the ability to link this code number with your personal information, and the linking information will be kept in on password-protected computers only accessible by the research team involved in this project. No information concerning the study or the data will be released to any third party, without prior approval by you and the written approval of the sponsoring institution. All participant interview audio recordings will be destroyed upon completion of the study. Your data will be stored for 15 years after the study finishes.

If you withdraw from the study, we will not collect any more information about you. We would like to keep the information we have already collected about you to help us ensure that the results of the research project can be measured properly. Please let us know if you do not want us to do this.

9. How will the results of the study be distributed?

It is anticipated that the results of this research project will be published and/or presented in a variety of forums. In any publication and/or presentation, information will be provided in such a way that you cannot be identified, except with your expressed permission.

You can indicate on the consent form if you wish to receive a lay summary of the study findings.

10. Who should I contact if I have any questions?

If you have any questions or want more information about this study before or during participation, you can contact:

Name: Prof Raghu Lingam

Phone: 0433 691 232

Email: r.lingam@unsw.edu.au

Name: Dr Carmen Crespo

Phone: 0406892209

Email: c.crespo@unsw.edu.au

11. Who do I contact if I have concerns about the study?

All research in Australia involving humans is reviewed by an independent group of people called a Human Research Ethics Committee (HREC). This study has been approved by the Sydney Children's Hospitals Network (SCHN) HREC **(approval number: 2022/ETH02068)**.

If you have any concerns or complaints about any aspect of the project or the way it is being conducted, you may contact the Executive Officer of the SCHN HREC on (02) 78251253 or SCHN-Ethics@health.nsw.gov.au.

This Information Sheet is for you to keep. We will also give you a copy of the signed consent form.

Parents/Carers-Consent Form
Implementation evaluation

**Study Title**	SUSTAIN Strengthening Care for Children (SUSTAIN)
**Principal Investigator/s**	Professor Raghu Lingam, Professor and Financial Markets Chair in Paediatric Population Health UNSW<br><br>Dr Annemarie Christie, Director Sydney Child Health Program, SCHN
**Main Study Contact Person**	Tammy Morris,<br>Research Associate<br>School of Clinical Medicine<br>University of New South Wales (UNSW)<br>**Email:** tammy_meyers.morris@unsw.edu.au<br>Phone: 0452494461

Declaration by Participant

☐ I have read the Participant Information Sheet or someone has read it to me in a language that I understand.

☐ I understand the purposes, procedures and risks of the research project described in the Participant Information Sheet.

☐ I have had an opportunity to ask questions and I am satisfied with the answers I have received.

☐ I freely agree to participate in this research project as described and understand that I am free to withdraw at any time during the project without affecting my / my child's future health care and relationship with SCHN or UNSW.

☐ I understand that I will be given a signed copy of this document to keep.

☐ I wish to receive a lay summary of the study findings via the following email / post address:

____

Name of Participant (please print): ____

Signature of Participant: ___________________________ Date: _______________________

Under certain circumstances (see Note for Guidance on Good Clinical Practice CPMP/ICH/135/95 at 4.8.9) a witness to informed consent is required.*

Name of Witness* to Participant Signature (please print): _________________________________

Signature of Witness: ______________________________________
Date: _______________________

* The Witness is not to be the investigator, a member of the study team or their delegate. In the event that an interpreter is used, the interpreter may not act as a witness to the consent process. Witnesses must be over 18 years of age
